# Bio-click chemistry: a bridge between biocatalysis and click chemistry

**DOI:** 10.1039/d1ra08053a

**Published:** 2022-01-12

**Authors:** Diego F. Rodríguez, Yanina Moglie, César A. Ramírez-Sarmiento, Sachin Kumar Singh, Kamal Dua, Flavia C. Zacconi

**Affiliations:** Facultad de Química y de Farmacia, Pontificia Universidad Católica de Chile Chile fzacconi@uc.cl; Departamento de Química, INQUISUR, Universidad Nacional del Sur (UNS)-CONICET Argentina; Institute for Biological and Medical Engineering, Schools of Engineering, Medicine and Biological Sciences, Pontificia Universidad Católica de Chile Santiago Chile; ANID – Millennium Science Initiative Program, Millennium Institute for Integrative Biology (iBio) Santiago Chile; School of Pharmaceutical Sciences, Lovely Professional University Phagwara 144411 Punjab India; Discipline of Pharmacy, Graduate School of Health, University of Technology Sydney NSW 2007 Australia; Faculty of Health, Australian Research Centre in Complementary and Integrative Medicine, University of Technology Sydney Ultimo Australia; Centro de Investigaciones en Nanotecnología y Materiales Avanzados, CIEN-UC, Pontificia Universidad Católica de Chile Santiago Chile

## Abstract

The fields of click chemistry and biocatalysis have rapidly grown over the last two decades. The development of robust and active biocatalysts and the widespread use of straightforward click reactions led to significant interactions between these two fields. Therefore the name bio-click chemistry seems to be an accurate definition of chemoenzymatic reactions cooperating with click transformations. Bio-click chemistry can be understood as the approach towards molecules of high-value using a green and sustainable approach by exploiting the potential of biocatalytic enzyme activity combined with the reliable nature of click reactions. This review summarizes the principal bio-click chemistry reactions reported over the last two decades, with a special emphasis on small molecules. Contributions to the field of bio-click chemistry are manifold, but the synthesis of chiral molecules with applications in medicinal chemistry and sustainable syntheses will be especially highlighted.

## Abstract 2

This review summarizes chemo/biocatalytic syntheses that combine the highly efficient click chemistry reactions with the advantages of biocatalysis. The concept of bio-click chemistry and its implications are introduced in this review.

## Graphical abstract

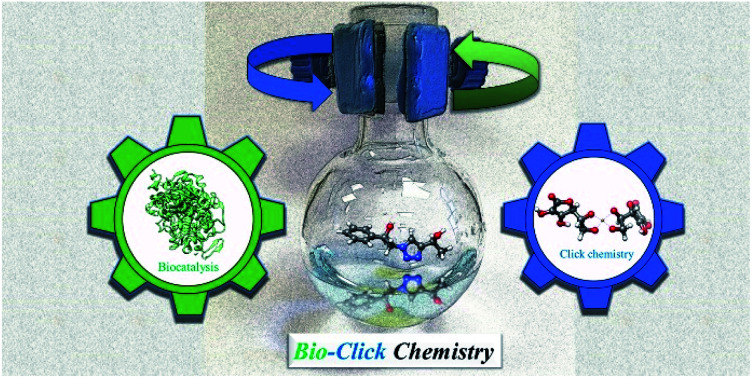

## 1. Introduction

Chemoenzymatic syntheses are powerful methodologies for the creation of complex molecular structures and functionalities.^1^ The development of processes which combine biocatalysis and chemical synthesis has rapidly increased over recent years. The two main pillars of innovation in the field are the generation of more active, selective, and stable biocatalysts,^2^ and the development of new synthetic procedures in water, allowing for highly efficient, and sustainable one-pot procedures.^3^

The term click chemistry was introduced by Sharpless in 2001, comprising highly efficient and reliable reactions enabling the rapid construction of structural and functional diversity through the union of small building blocks. Commonly these reactions have very high inherent driving forces, thus avoiding tedious chromatographic work-up.^4^ Evidently, the concept of click chemistry is largely inspired by nature's “synthetic toolbox”, and it is not surprising that the merger of click chemistry and biocatalysis exhibits great potential for the synthesis of complex molecular structures.

**Fig. 1:**
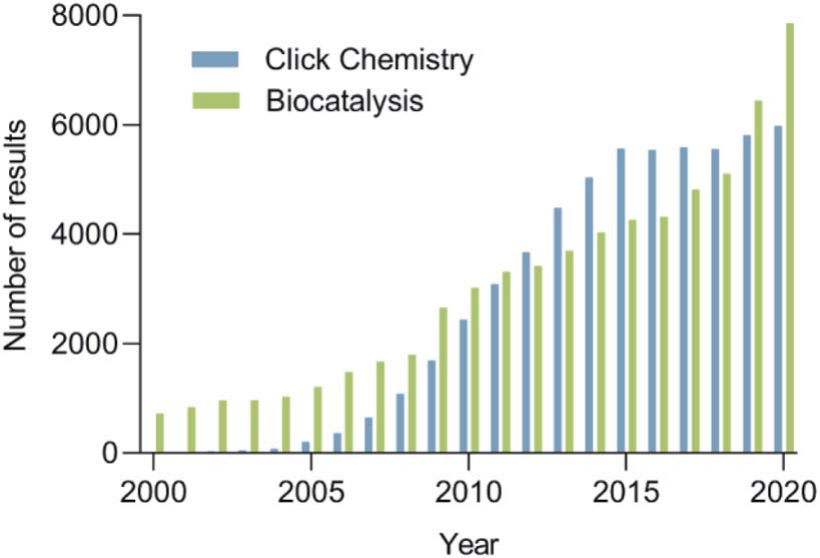
The number of results to the search for click chemistry (blue) and biocatalysis (green) in Scopus per year between 2000–2020.

The increasing demand for sustainable processes is fostering the development and establishment of new synthetic tools. Among these tools, click chemistry and biocatalysis are of utmost importance, since they are characterized by their high selectivity and orthogonality, mild reaction conditions avoiding protecting groups, efficiency, reliability, and user-friendliness.^5^ The growing interest in click chemistry and biocatalysis in recent years is reflected by the drastic increase in the number of publications on the topic (Fig. 1). It is not surprising that the combination of these two disciplines, which we propose to call bio-click chemistry, has given rise to a myriad of highly interesting developments and has led to a significant impact on the synthesis of structurally diverse molecules, such as chiral amino alcohols, triazoles, amides, cycloadducts, thioethers, and others. In addition, bio-click methodologies show a synergy oftentimes allowing for shorter, stereoselective and efficient synthetic routes.

The present review focuses on syntheses in which biocatalytic processes and click reactions were used in combination. Recent reviews have focused on specific applications of click chemistry and biocatalysis in the *in situ* generation of inhibitors,^6,7^ the immobilization and modification of enzymes,^8^ or the generation of semisynthetic enzymes.^9^ However, the discrete topic of bio-click chemistry has yet to be approached in depth. We seek to fill this gap and discuss the most important aspects of the development of bio-click methodologies, as well as their main advantages and limitations.

## 2. Bio-click chemistry

**Fig. 2:**
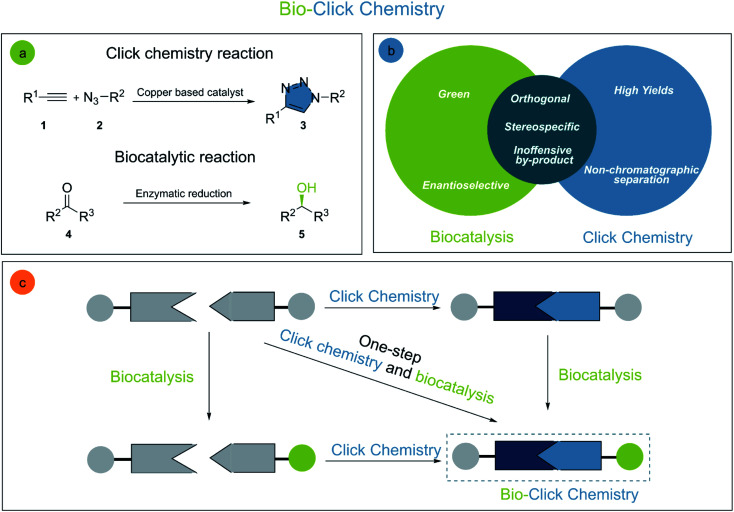
(a) General click chemistry and biocatalysis reactions, (b) bio-click chemistry interface and (c) schematic representation of bio-click chemistry cooperation for the synthesis of small molecules.

Organic chemistry has a sheer endless set of reactions allowing for the generation of molecular diversity and complexity. Chlorophyll,^10^ taxol,^11,12^ and human lysozyme^13^ are only a few examples of highly important complex molecules obtained through elegant chemical syntheses. Albeit highly sophisticated, organic synthesis is undergoing constant improvements^14^ especially through the development of more selective, efficient, and sustainable reactions. The interconnection of different disciplines, such as biocatalysis and click chemistry, is essential to achieve these goals (Fig. 2).

Click chemistry is generally outlined by a group of reactions with desirable characteristics for biocompatible reactions or bioconjugations: reliability, effectiveness, protecting group-free, high yielding, and easy to purify (often without any chromatography). The fact that click chemistry has become an integral part of the synthetic toolbox is demonstrated by its success in chemical synthesis, drug discovery, medicinal chemistry,^15^ molecular labelling,^16^ polymer synthesis,^17^ and others.

The cutting-edge example for click chemistry is (i) the copper-catalyzed 1,3-dipolar cycloaddition of terminal alkynes and organic azides (CuAAC). Nevertheless, many other transformations are classified as click reactions as well (ii) the Diels–Alder reaction, (iii) additions to alkenes, (iv) nucleophilic opening of strained rings, (v) non-aldol carbonyl transformations, and (vi) addition reactions to alkynes.^18^ It has to be clarified, that although click reactions are regio- and stereospecific, they are not necessarily enantioselective.^4^ This shortcoming can be overridden by the merger of click chemistry with enantioselective biocatalysts such as enzymes.

Biocatalysis comprises the use of purified enzymes, cell-free extracts or whole microorganisms as catalysts in a vast array of transformations, rendering them as excellent and sustainable methods for the construction of complex molecular structures, while fulfilling green chemistry principles.^19^ However, enzymes have inherent limitations often preventing their widespread use in synthesis: rather low operational stability, inhibition by substrates or products, limited tolerance of non-natural substrates and limited applicability in organic solvents. Innovative technologies, such as protein engineering and enzyme immobilization, have emerged to overcome these drawbacks and limitations.^2^ Many enzymes obtained by protein engineering are more robust, stable and capable to transform non-natural substrates. Immobilized enzymes generally present outstanding operational stability, an easy removal from the reaction medium and the capability of repetitive recycling.

Enzymatic processes allowing for the synthesis of complex molecules under highly sustainable and green conditions are increasingly popular. Various drug molecules commonly synthesized through traditional catalytic processes are eventually produced by chemoenzymatic methods, thus improving process efficiencies and substantially reducing waste generation.^20,21^

**Fig. 3:**
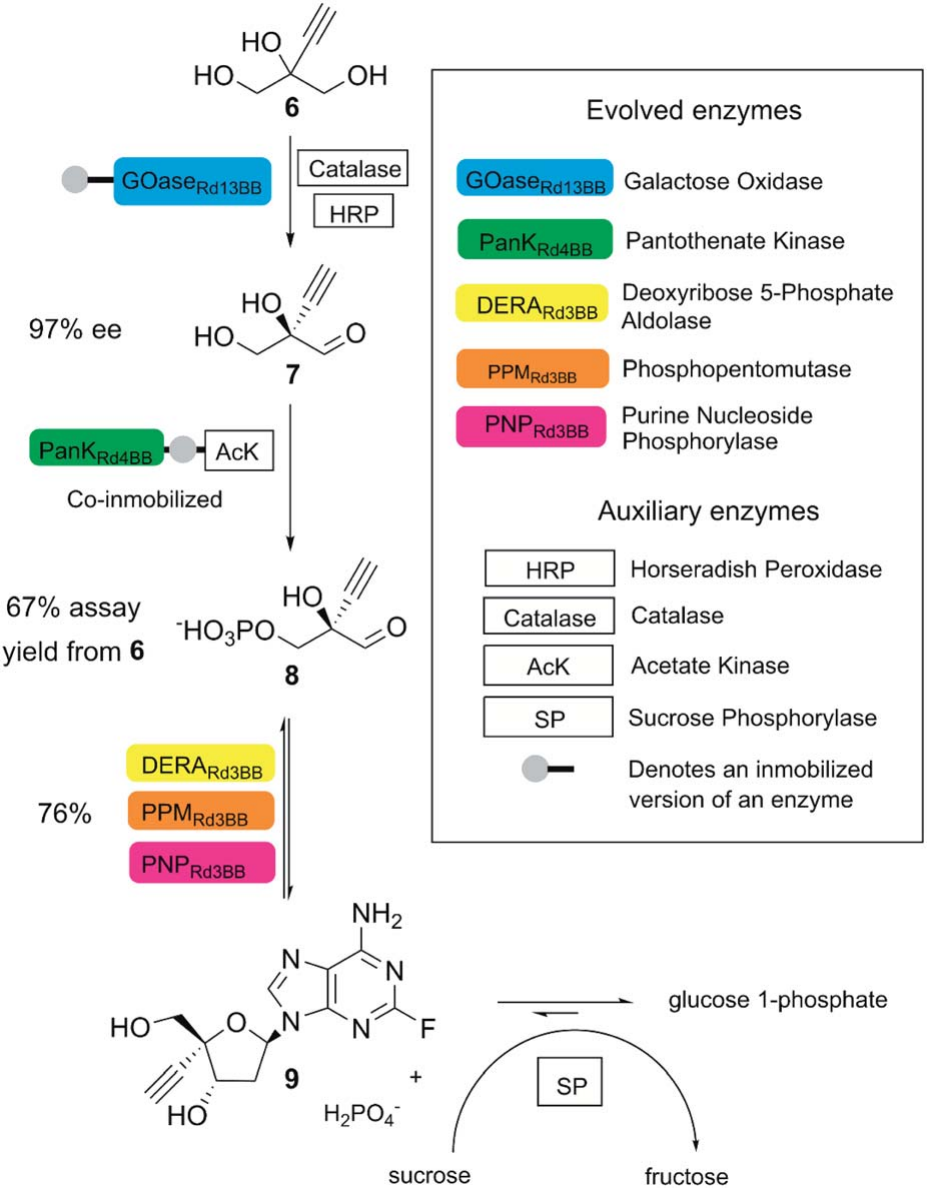
Biocatalytic synthesis of islatravir. (This figure has been adapted from ref. 22. With permission from Science Journal, copyright 2020).

A state-of-the-art example is the biocatalytic synthesis of the HIV treatment candidate islatravir, as reported by Huffman and co-workers.^22^ In this process, five enzymes obtained by directed evolution (two immobilized enzymes) and four auxiliary enzymes were applied towards the generation of islatravir from simple achiral building blocks in a three-step cascade reaction (Fig. 3). The target molecule was obtained in 51% overall yield, with high atom economy, using water as solvent, avoiding intermediate purification and in less than half of the number of steps compared to the conventional chemical synthesis.^22^

Although, Caruso *et al.* used the term bio-click chemistry in a study towards the functionalization of polymer drug vehicles,^23^ a more exhaustive review of the interface between click chemistry and biocatalysis is necessary. Considering the potential for the development of highly efficient, sustainable, and selective processes, a profound impact of bio-click methods on the field can be envisaged.

It should be noted that the term “bio-click chemistry” differs markedly from click chemistry, as it interconnects the latter with biocatalytic processes. In many cases, this allows for the development of greener processes. However, it must be clarified that the examples covered in this review are based not only on sustainability or higher yields, but rather cover a broad spectrum of cases allowing us to show the current advantages and limitations of bio-click chemistry.

## 3. Bio-click reactions

Click chemistry is a dynamic and growing field, continually leading to new transformations. Many of these developments are predominantly focused on bioconjugation and applications in materials science.^24^ Examples for new transformations characterised as click reactions are the sulfur(vi) fluoride exchange (SuFex)^25^ and the synthesis of azides from primary amines^26^ respectively. Nevertheless, interactions of the latter with biocatalysis for the creation of new molecules are still missing. Significant cooperation between click chemistry and biocatalysis has been reported for CuAAC-, Diels–Alder-, epoxide-opening- and thiol-Michael reactions, and these studies will be discussed in further detail.

### 3.1 CuAAC and biocatalysis

**Scheme 1:**
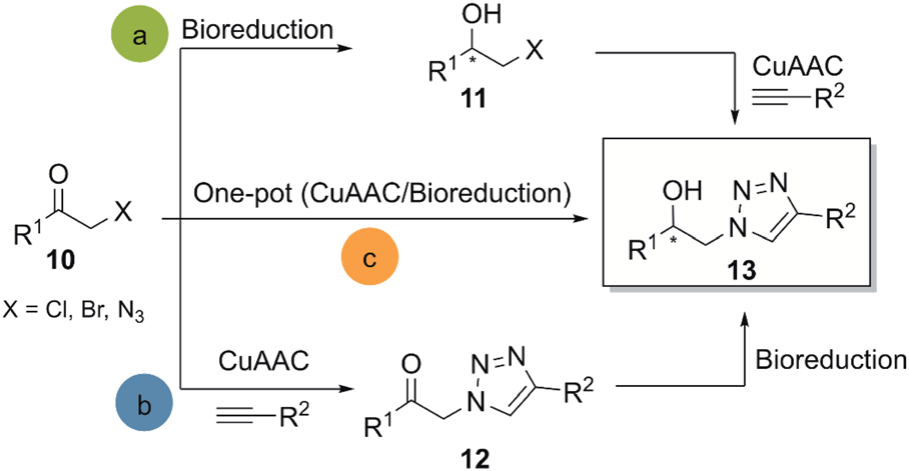
General methodologies for the chemo-biocatalytic synthesis of β-hydroxytriazoles.

CuAAC is probably the most representative click reaction. It allows for the synthesis of 1,2,3-triazoles from organic azides and alkynes in the presence of a copper-(i)-catalyst (Scheme 1). The 1,2,3-triazole ring has shown great value as a pharmacophore, and several reviews focused on the potential of this invaluable motif in medicinal chemistry.^15,27^ Regarding the bio-click approach, the main focus in literature is the combination of CuAAC with oxidoreductases and hydrolases respectively.

#### 3.1.1 CuAAC and oxidoreductases

The most common application of click reactions combined with oxidoreductases is for the synthesis of highly enantiopure β-hydroxytriazoles 13. These compounds present excellent pharmacological properties, as they are able to act as potential HIV protease inhibitors^28^ and β-adrenergic receptor blockers.^29^ Enantiopure β-hydroxytriazoles can be generally obtained by three distinct approaches: (a) bioreduction of α-chloro/bromo-acetophenones or α-azidoacetophenones with subsequent CuAAC reaction, (b) the generation of β-ketotriazoles and subsequent bioreduction, (c) a simultaneous one-pot bioreduction and triazole formation (Scheme 1).

**Scheme 2:**
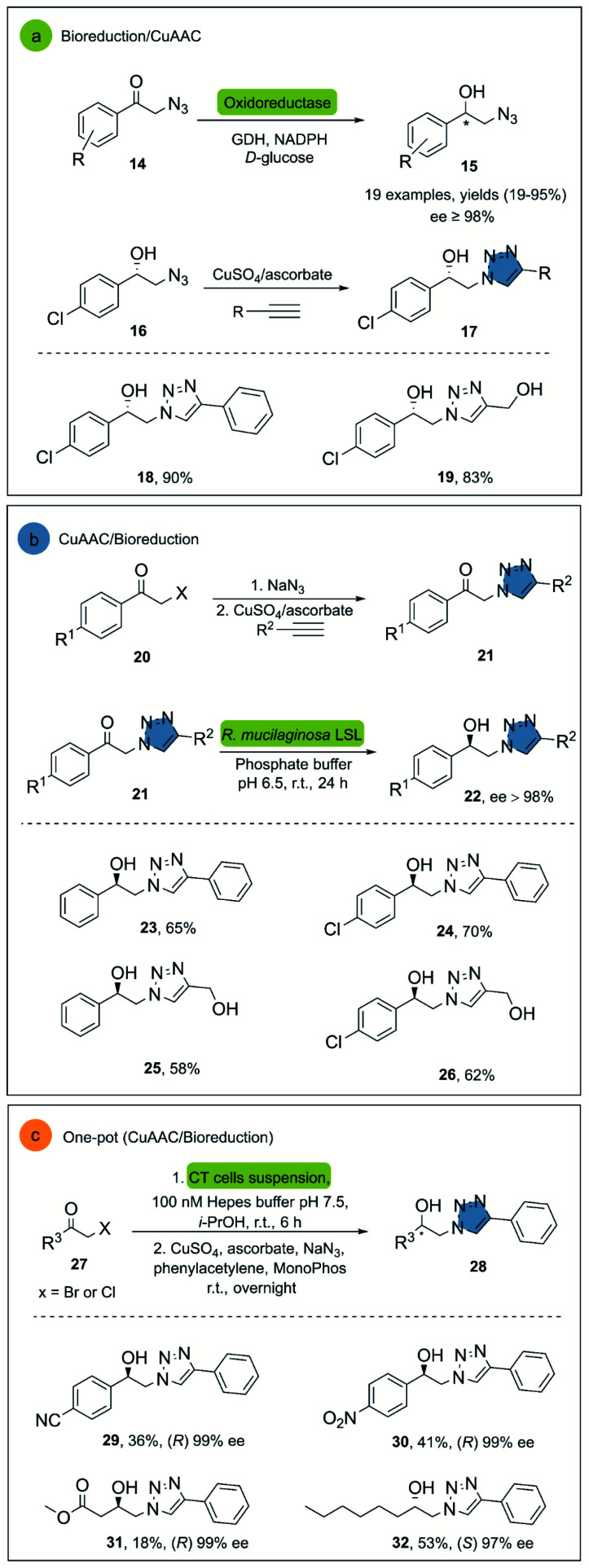
(a) Synthesis of β-blocker analogues containing 1,2,3-triazoles moieties, (b) stereoselective reduction of β-ketotriazoles with *R. mucilaginosa* LSL, (c) one-pot whole-cell catalysis and CuAAC for the conversion of α-haloketones to β-hydroxytriazoles.

In 2008, Hua and co-workers reported the use of a recombinant carbonyl reductase from *Candida magnoliae* (CMCR) and an alcohol dehydrogenase from *Saccharomyces cerevisiae* (Ymr226c), with glucose dehydrogenase as cofactor-regeneration enzyme, for the enzymatic reduction of α-azidoacetophenone.^29^ Both enzymes were able to catalyze the formation of the corresponding chiral alcohol in excellent optical purities, good yields and with wide substrate scope. After the successful bioreduction of 2-azidoacetophenones 14, the desired 1,2,3-triazoles 17 were obtained in high yields, using CuSO_4_ and sodium ascorbate as catalytic system (Scheme 2a).

Another interesting example is the direct synthesis of enantioenriched β-hydroxytriazoles from the corresponding haloketones and β-ketotriazoles.^30^ In this approach, a whole-cell procedure of wild type *Rhodotorula mucilaginosa* LSL successfully catalyzed the one-pot enantioselective bioreduction of the substrate in water at room temperature, although only four examples were reported under these conditions (Scheme 2b). Notably, the reactants necessary for the ketotriazole formation, such as sodium azide and copper sulfate, did not affect the *R. mucilaginosa* LSL system in its catalytic activity. This represents an important advantage, avoiding purification steps and the manipulation of organic azides, often exhibiting explosive properties.^31^

Janssen and co-workers described an alternative system for the synthesis of β-hydroxytriazoles using a one-pot methodology.^32^ The developed system consists of the *E. coli* strain MC1061, which overexpresses enzymes such as alcohol dehydrogenases (ADH) and halohydrin dehalogenases (HHDH) with different stereospecificities. The construct that overexpressed (*R*)-stereospecific (ADH) and (HHDH) was called CT-cells and produce (*R*)-enantiomers, while the BT-cells form the (*S*)-enantiomers in high enantiomeric purity, albeit only moderate yields. The catalyst for the click reaction was CuSO_4_ with sodium ascorbate in the presence of MonoPhos as ligand, increasing the CuAAC reaction rate^33^ (Scheme 2c).

**Scheme 3:**
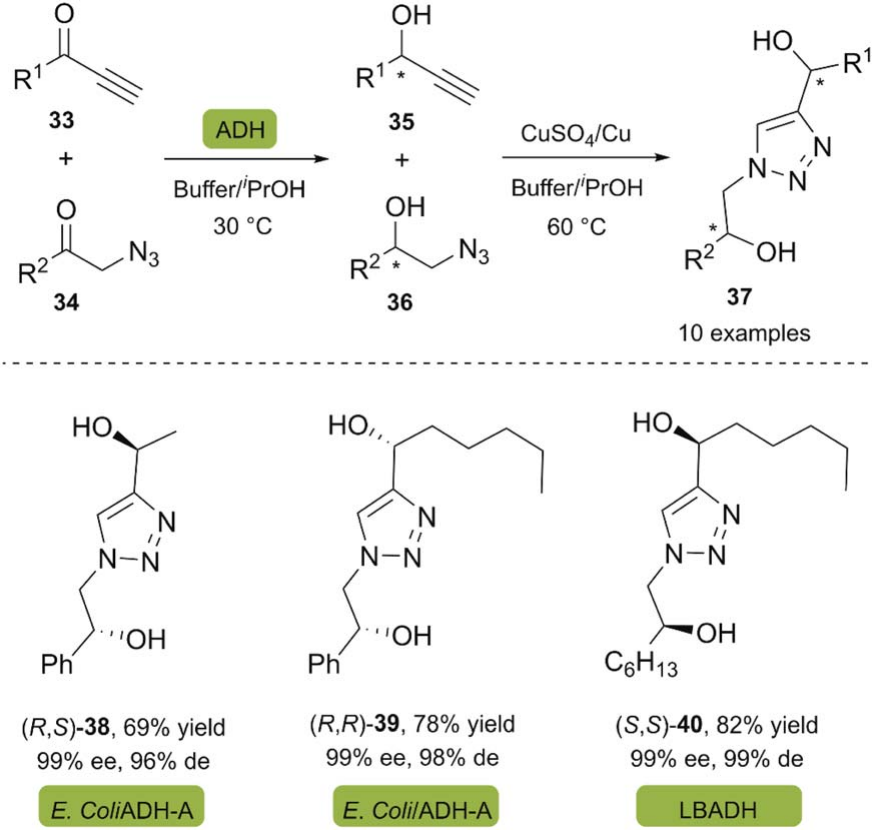
Synthesis of dihydroxytriazoles from chemoenzymatic approach.

Chiral diols are a group of compounds widely used as organocatalysts and intermediates for the preparation of chiral heterocycles.^34,35^ Gotor and co-workers reported the synthesis of dihydroxytriazoles 37 through a highly efficient chemoenzymatic procedure under mild reaction conditions.^36^ In this two-stage one-pot procedure, the recombinant enzyme ADH-A from *Rhodococcus rubber* overexpressed in *E. coli* (Prelog-enzyme) and a commercially available LBADH from *Lactobacillus brevis* (anti-Prelog-enzyme) were used for the bioreduction of several alkynones 33 and α-azido ketones 34 in a phosphate buffer with 2-propanol. A copper wire was used as a reusable catalyst for the click reaction, supplemented by a catalytic amount of copper sulfate (Scheme 3).

**Scheme 4:**
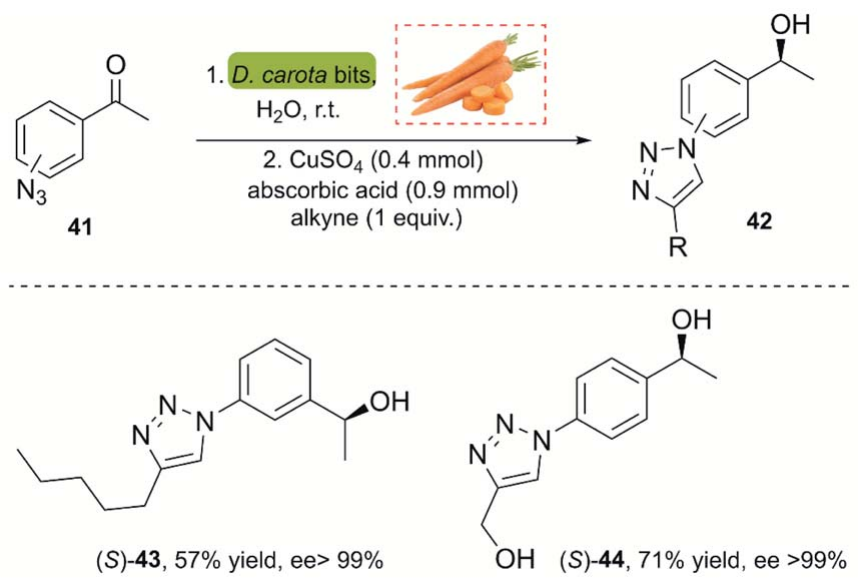
A chemoenzymatic methodology for the synthesis of chiral hydroxytriazole.

A powerful methodology for asymmetric ketone reduction was reported by Omori and co-workers in 2013, using whole cells from plant tissue from carrots (*Daucus carota*). The main advantage of this approach is its simplicity, economy of the process and the easy availability. The reaction features the one-pot bioreduction of azidoacetophenones (*p*,*m*-substituted) 41 with the subsequent CuAAC reaction using the Sharpless–Fokin catalyst (Scheme 4).^37^ The products were obtained in moderate yields but excellent enantiomeric excess (>99%). However, a disadvantage of this process is the rate of the bioreduction, requiring 5–7 days for completion. This impedes the implementation of a single-stage one-pot procedure, since the click reaction occurs very fast, and non-reduced triazoles are insoluble in water.

**Scheme 5:**
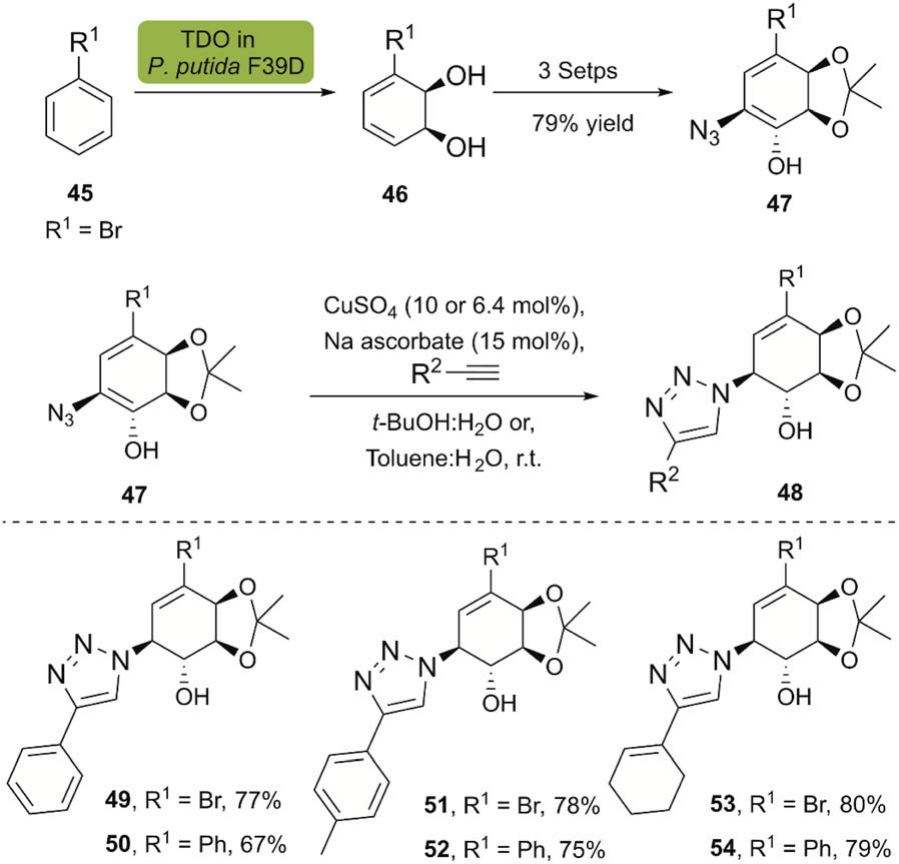
Chemo-enzymatic synthesis of the bromoazidoconduritol derivative, and synthesis of triazolylconduritols.

Dioxygenases are versatile biocatalysts that have been used in the chemoenzymatic synthesis of chiral hydroxyazides. In 2011, the Stefani group reported the enzymatic dihydroxylation of arenes 45 catalyzed by whole-cell *P. putida* F39/D en route to chiral azides 47. The subsequent click reaction between 47 and several alkynes was achieved with the Sharpless–Fokin catalyst in *t*-BuOH : H_2_O or toluene : H_2_O,^38^ and the corresponding triazolylconduritols 48 were obtained in good to excellent yields (Scheme 5).

#### 3.1.2 CuAAC and hydrolases

**Scheme 6:**
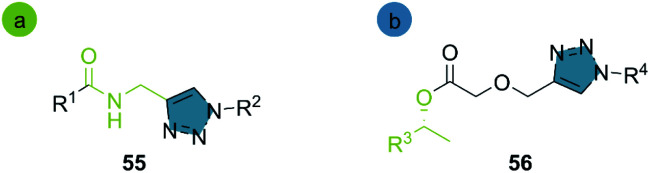
Representative structures obtained by chemoenzymatic hydrolases/CuAAC reactions in (a) peptidomimetic synthesis (b) kinetic resolution of racemates.

Over the last decade, chemoenzymatic methods based on the combination of hydrolases and the click CuAAC reaction have been successfully implemented for peptide-triazole synthesis 55 and the kinetic resolution (KR) of racemic secondary alcohols 56 (Scheme 6).

Enzymatic aminolysis is an excellent environmentally-friendly alternative to coupling reactions generally used in amide synthesis, and its importance is expected to increase over the coming years.^39^ However, the enzymatic formation of the peptide bond usually requires a high amount of biocatalyst, which is a major drawback. The sustainability of these processes was greatly improved with the implementation of immobilized enzymes, which are usually very stable and easily removed from the reaction medium, playing a crucial role in green amide syntheses.

Due to their structural and electronic similarity, 1,2,3-triazoles can act as peptidomimetic amides. Specifically, the 1,4-disubstituted 1,2,3-triazoles are bioisosteres of *trans*-amide compounds.^40^ Due to the importance of peptides as pharmacologically active compounds, the triazol-ring represents an excellent alternative for the synthesis of peptides with increased metabolic stability, as well as biologically-active natural product analogs difficult to obtain through conventional synthesis.

**Scheme 7:**
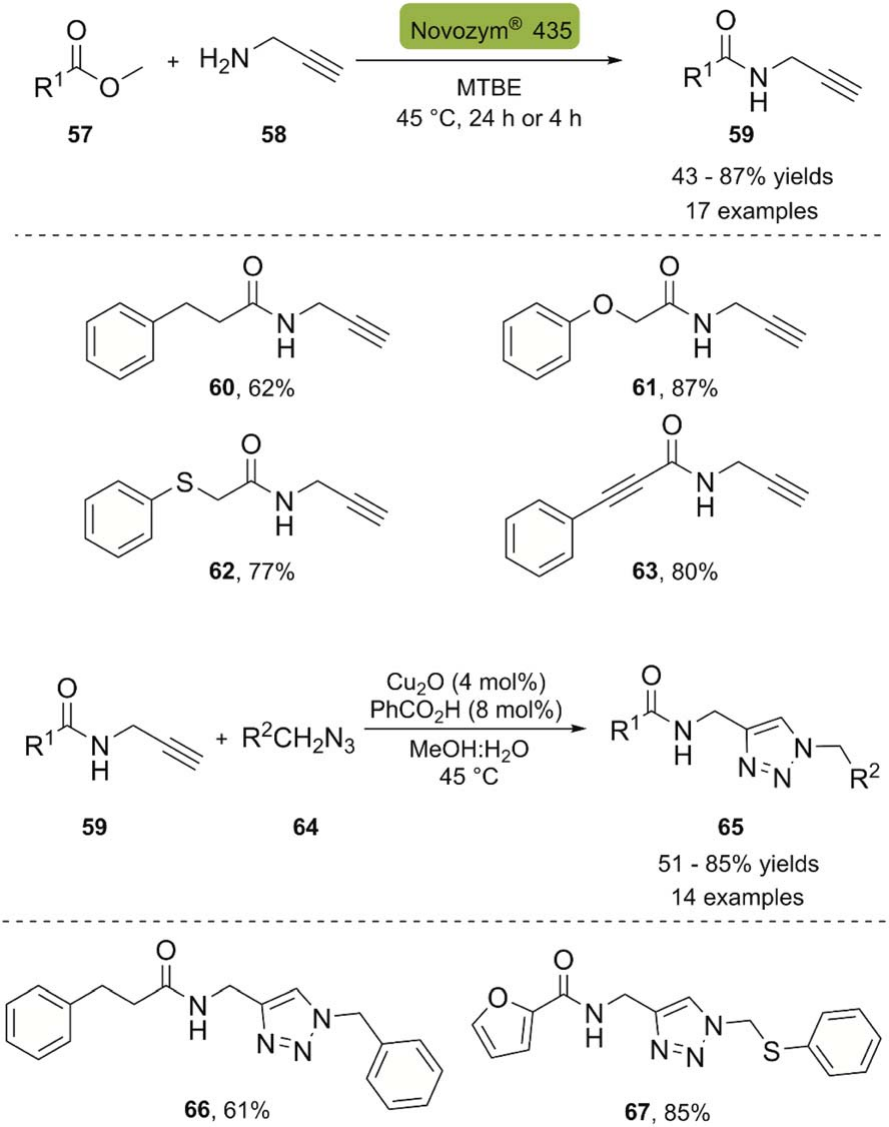
Synthesis of propargyl amides with CAL-B (Novozym® 435) and consecutive three-component synthesis of the amido methylsubstituted 1,2,3-triazoles.

In 2013, the Müller group reported the first enzymatic aminolysis catalyzed with lipases and a subsequent CuAAC reaction.^41^ For the aminolysis reaction, different commercially available immobilized lipases were studied. However, only Novozym® 435 and Immobead® 150 successfully catalyzed the reaction of methyl esters 57 with propargyl amine 58. Novozym® 435 (*Candida antarctica* lipase B [CALB] immobilized in Lewatit VP OC 1600) proved to be superior and achieved 68% conversion in 24 h. The optimized reaction conditions can be applied to a broad range of substrates and generally achieve good yields. The biocatalytic synthesis of propargylamides was then coupled to the CuAAC reaction using CuO_2_/benzoic acid as catalyst, and H_2_O : MeOH (1 : 1) as solvent system. The amide-triazole products 66–67 were obtained with yields between 51–85% (Scheme 7).

**Scheme 8:**
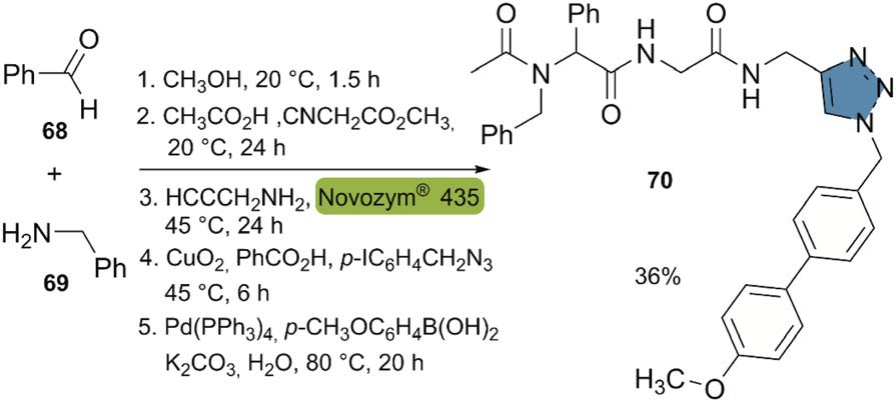
Consecutive seven-component sequence: Ugi reaction, CALB-catalyzed aminolysis, CuAAC, Suzuki coupling for the synthesis of product 70.

Based on the aforementioned studies, the Müller group reported the consecutive seven-component synthesis of triamides with a triazole moiety in 2019.^42^ This approach incorporated the biocatalytic aminolysis-CuAAC reaction in a new 5-stage multicomponent one-pot synthesis. The reactions featured (i) the synthesis of a diamide with a methyl ester group, from a 4-component Ugi-reaction, (ii) the propargylamide synthesis catalyzed by Novozym® 435, (iii) a CuAAC reaction and (iv) a Suzuki cross-coupling to the final product. This sequential one-pot procedure allowed to synthesize compound 70 in 36% overall yield. The described method allows for the generation of compound libraries, in an easy way without the need for intermediate purification (Scheme 8).

**Scheme 9:**
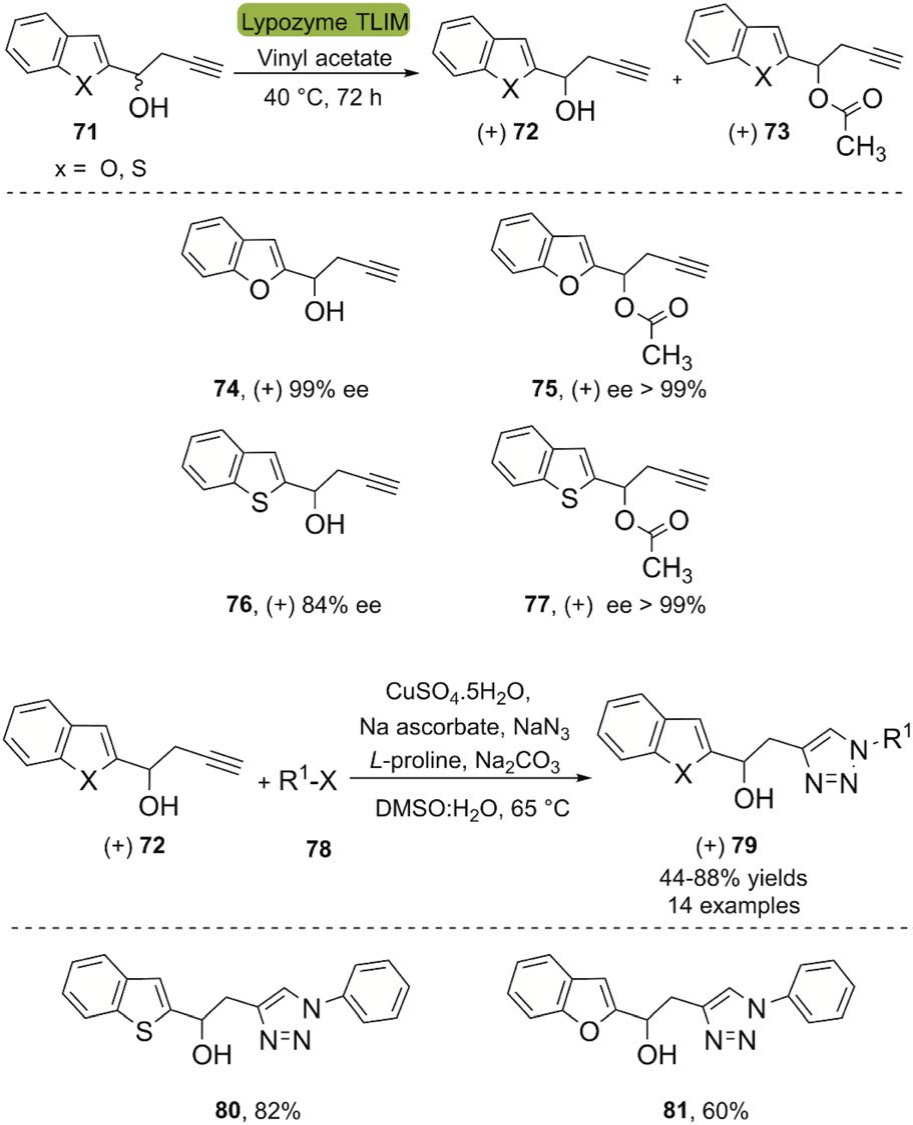
Enzymatic resolution of racemic homopropargylic alcohols with TLIM and multicomponent synthesis of 1,4-disubstituted 1,2,3-triazole derivatives.

A second application of the hydrolases combined with CuAAC are the kinetic resolutions of racemic mixtures. In this context, Büyükadali *et al.* described the one-pot synthesis of chiral benzothiophenyltriazoles and benzofuranyltriazoles in 2015.^43^ This bio-click approach produces the enantiomerically enriched homopropargylic alcohols (+) 72 using the commercially available TLIM (immobilized *Thermomyces lanuginosus* lipase). TLIM proved superior to other lipases, such as Novozym® 435 concerning both, enantioselectivity and reaction time. Furthermore, the reaction occurs in vinyl acetate, which acts as an acyl donor and solvent (Scheme 9). Unfortunately, the amount of biocatalyst necessary was 1 : 1% w/w with respect to the substrate, and recycling experiments of the catalyst were not reported. The concatenation of the TLIM-mediated enzymatic resolution and a click reaction was achieved through a subsequent one-pot multicomponent CuAAC reaction using CuSO_4_·5H_2_O/Na ascorbate, NaN_3_, l-proline, and Na_2_CO_3_, furnishing the corresponding triazole derivatives (+) 79 with good yields (Scheme 9). The *in situ* generation of the organic azide eliminates the need of an intermediate purification process.

**Scheme 10:**
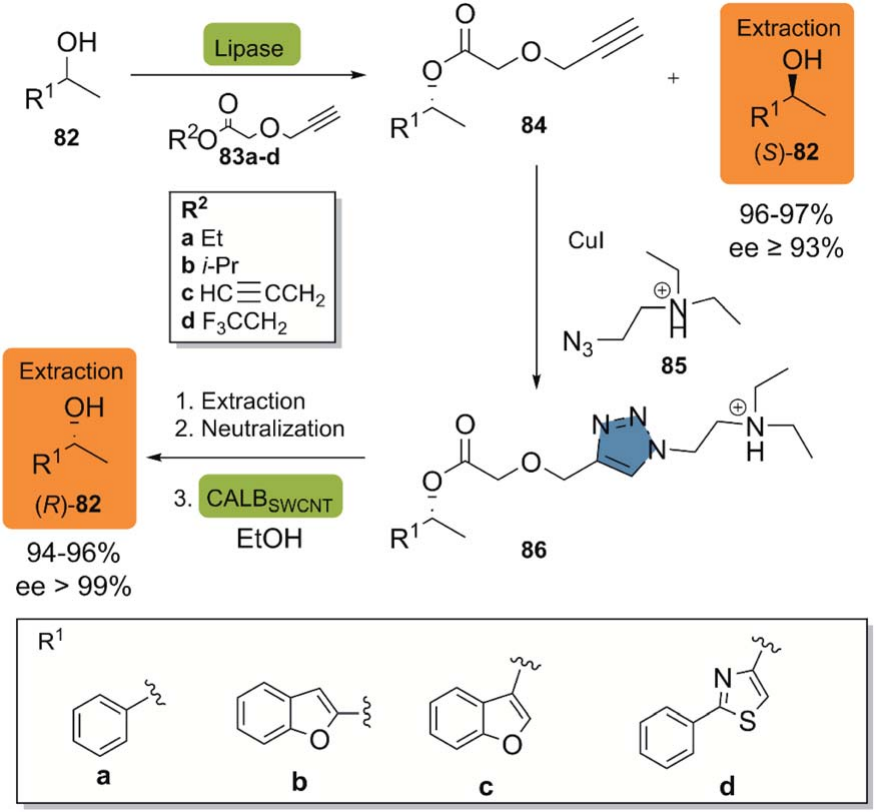
Production of the two enantiomeric forms of (hetero)arylethanols by an enzymatic KR/click reaction-based separation process. This figure has been Adapted from ref. 44 with permission from the Royal Society of Chemistry.

In 2018, Moisă *et al.* reported a new bio-click approach for the separation of racemic secondary alcohols 82 by simple extraction.^44^ This methodology uses the enantioselectivity of the lipases of Novozym® 435 and *Pseudomonas fluorescens* (L-AK) for the catalysis of an O-acylation of heteroarylethanols with different esters 83a–d as acylating agents. The corresponding (*R*)-product 84 is then subjected to a CuAAC reaction with azido-functionalized tertiary amine 85 in the presence of CuI, furnishing the triazole quantitatively. The ionizable triazole ester was efficiently extracted with an aqueous acetic acid solution, while the (*S*)-82 alcohol was recovered from the organic phase. The alcoholysis of the triazole derivative in the presence of ethanol was catalyzed by CALB_SWCNT_ (CALB covalently immobilized on single-walled carbon nanotubes) and allowed for the obtention of the (*R*)-82 alcohol (Scheme 10). Up-scaling of the process to a multi-gram scale revealed, that there was neither a change in the selectivity of the enzymes nor in the efficiency, as reflected in virtually unchanged isolated yields of the products (>99%).

### 3.2 Diels–Alder reactions and biocatalysis

**Scheme 11:**
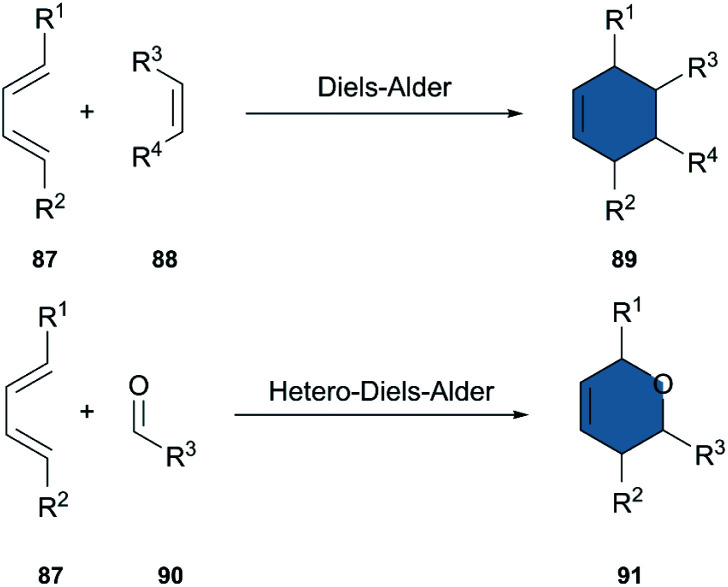
Diels–Alder reaction and hetero-Diels–Alder reaction.

The Diels–Alder (DA) reaction is an outstanding tool for the synthesis of cyclohexenes and other ring structures. Involving the cycloaddition of a diene and a dienophile, it has been extensively used for the synthesis of complex molecules and is one of the most powerful transformations in organic chemistry (Scheme 11). Moreover, the DA reaction is stereospecific, shows a high atom economy and reliability, and can be carried out in environmentally-friendly solvents.^45,46^ A hetero-Diels–Alder-reaction (HAD) is equally well studied, and is a reliable alternative for the synthesis of ring structures including heteroatoms.

In contrast to the CuAAC reaction, the DA reaction can be carried out without a catalyst. However, the use of Lewis acids allows to decrease the activation energy of the DA reaction, increasing its rate and lowering the necessary reaction temperature.^47–49^ A further increase of the reaction rate can be achieved by the incorporation of EWG-groups in the dienophile and EDG-groups in the diene, favoring the electronic distribution necessary for the reaction to occur.

#### 3.2.1 DA/HDA and oxidoreductases

**Scheme 12:**
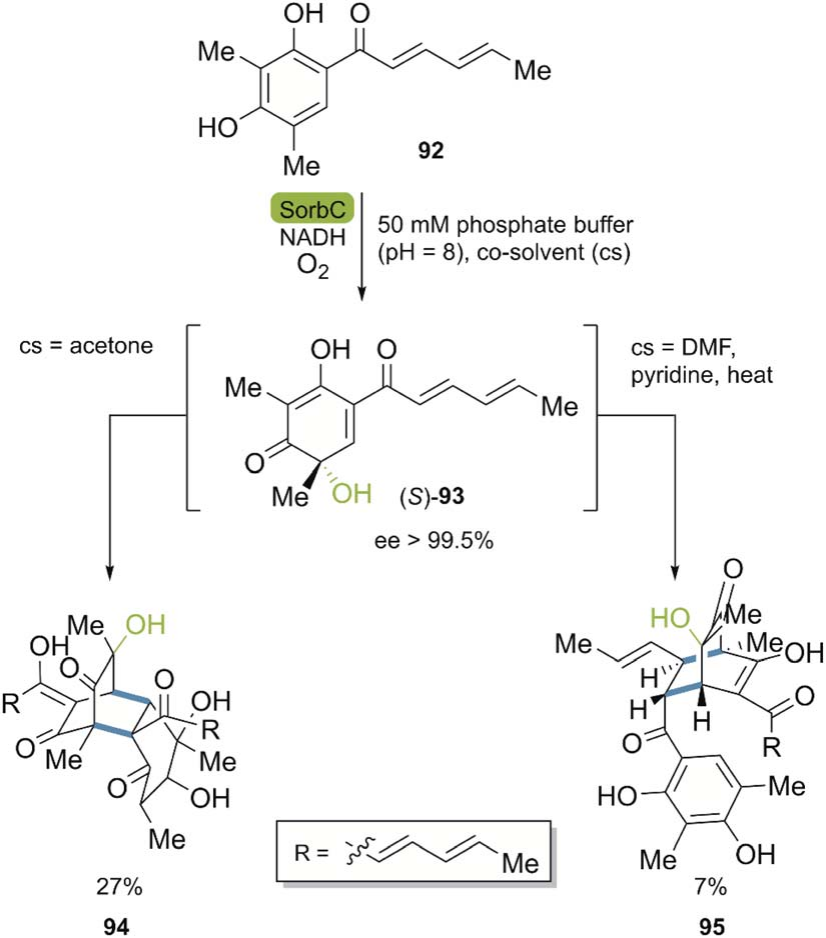
Key step of the stereoselective enzymatic total synthesis of bisorbicillinol 94 and sorbiquinol 95.

The combination of DA reactions with oxidoreductases has been successfully applied in the stereoselective synthesis of complex molecules. The bio-click approach has been used for the synthesis of sorbicillin derivates, an important family of compounds from fungi, exhibiting promising biological activities.^50^ In 2017, the Gulder group reported the total synthesis of bisorbicillinoids by an enzymatic oxidative dearomatization of sorbicillin 92 and a subsequent DA reaction (Scheme 12).^51^ In the first part of the synthesis, the enzyme SorbC catalyzed the enantioselective formation of sorbicillinol (*S*)-93 (ee > 99.5%), followed by the rapid dimerization to the product through a DA reaction. The solvent polarity proved to be crucial for the stability of sorbicillinol (*S*)-93, when CH_2_Cl_2_ was used to quench the reaction, the bisorbicillinol 94 was obtained in a 27% yield in 40 minutes, without any other dimeric analogs. Different co-solvents could be used for the control of the selective formation of different byproducts. As an example, the use of DMF combined with the work-up comprising extraction with CH_2_Cl_2_, a fast evaporation of CH_2_Cl_2_ after 4 h, and the subsequent heating in the presence of pyridine furnished sorbiquinol 95 in 7% yield. This chemoenzymatic approach proved to be an excellent alternative in comparison to the chemical syntheses reported to date. For example, the Deng group reported an elegant enantioselective total synthesis of bisorbicillinol 94 with a global yield of 27%, however applying 10 steps.^52^

**Scheme 13:**
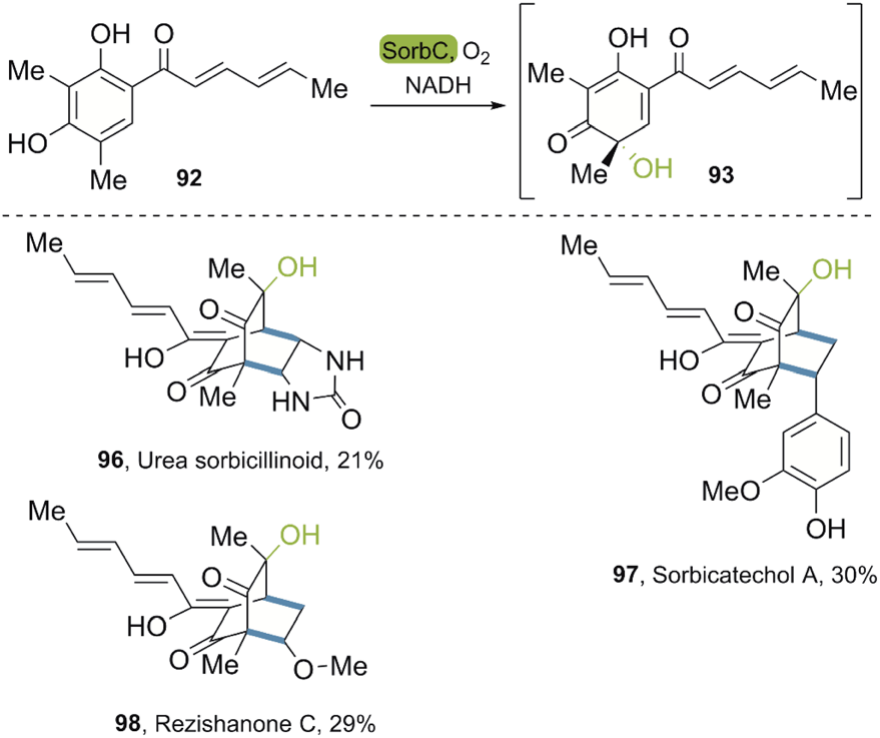
Chemoenzymatic synthesis of the sorbicillinoids *via* enantioselective oxidative dearomatization of sorbicillin with SorbC and DA reaction.

In 2018, the groups of Gulder and Narayan independently reported a chemoenzymatic approach towards the synthesis of new sorbicillin derivates.^53,54^ In these investigations, the use of the SorbC enzyme once again furnished sorbicillinol 93 by oxidative dearomatization as a key step. The use of CH_2_Cl_2_ enabled the extraction of 93; and its subsequent reaction with different dienophiles allowed the production of sorbicillin derivates in a simple chemoenzymatic process. The scope of products was very versatile, giving access to urea sorbicillinoid 96, sorbicatechol A 97, and rezishanone C 98 (Scheme 13).

The Narayan-group synthesized the urea sorbicillinoid 96*via* a DA reaction between sorbicillin and bisacylated urea and following by the addition of LiOH. Furthermore, they described different monooxygenases, such as Trop B and AzaH, for the catalysis of the oxidative dearomatization of resorcinols, allowing for the synthesis of valuable *ortho*-quinol products,^54^ a process that was adapted to gram scale.

#### 3.2.2 DA/HDA and hydrolases

**Scheme 14:**
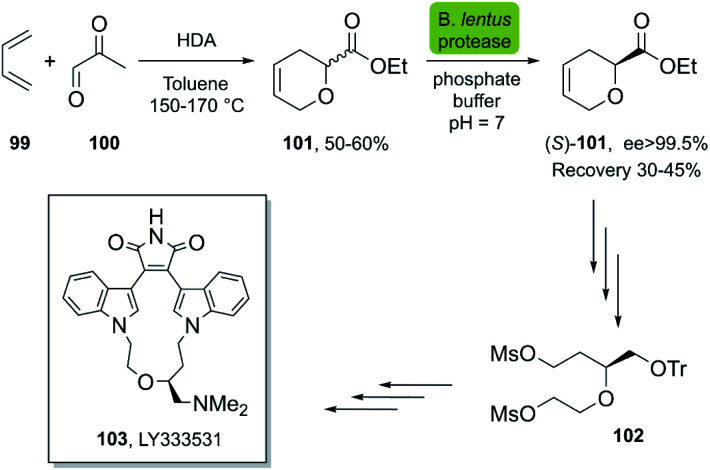
Chemoenzymatic synthesis of (*S*)-101, a key intermediate in the synthesis of LY333531, a protein kinase C inhibitor (PKC β).

In 2002 Caille *et al.*^55^ reported the chemoenzymatic synthesis of ethyl (*S*)-3,6-dihydro-2*H*-pyran-2-carboxylate 101, which is a useful intermediate in the synthesis of macrocyclic bisindolylmaleimide (LY333531) 103, a potent inhibitor of protein kinase Cβ (PKC β).^56^ The bio-click approach developed by Caille *et al.* took advantage of inexpensive starting materials, namely butadiene 99 and ethyl glyoxylate 100, producing 2-carboethoxy 3,6-dihydro-2*H*-pyran 101 in a HAD-reaction. The subsequent resolution of the racemic ester by selective enzymatic hydrolysis using *B. lentus* protease was carried out on a gram-scale, and after 8 h the ee of the (*S*)-configured ester was >99.5% (Scheme 14).

**Scheme 15:**
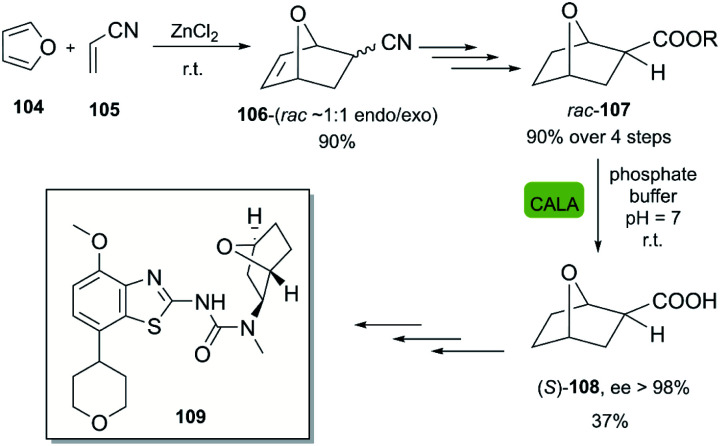
Bio-click approach for the synthesis of (*S*)-108. Precursor in the synthesis of A2 receptor antagonist 109.

In 2010, Wirz *et al.* reported the scalable enantioselective synthesis of a benzothiazole derivative 109, which was evaluated as an A2 receptor antagonist for the treatment of major depression.^57^ The developed chemoenzymatic process consists of the DA-reaction of furan 104 with acrylonitrile 105, activated by ZnCl_2_, furnishing bicyclic product 106. The following steps gave rise to the racemic product 107, which was hydrolyzed by CALA lipase in a stereoselective fashion, furnishing the (*S*)-enantiomer 108 with excellent enantioselectivity (Scheme 15).

**Scheme 16:**
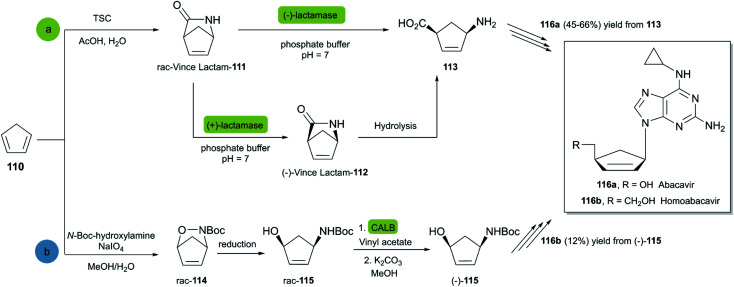
Chemoenzymatic synthesis of abacavir. (a) Using a lactamase by kinetic resolution of *rac*-Vince lactam and (b) using a CALB lipase in the kinetic resolution of aminocyclopentenol derivate.

Abacavir is a carbocyclic nucleoside with antiviral activity, acting as a reverse transcriptase inhibitor (see Scheme 16). It is used in combinations with other nucleoside analogues, such as lamivudine, for the treatment of HIV-infection.^58^ Crimmins *et al.* described an asymmetric synthesis of abacavir in 1996, making use of a first-generation Grubbs catalyst.^59^ An alternative synthetic route (Scheme 16a), used the Vince lactam 111, which can be obtained by the DA reaction of cyclopentadiene 110 and tosyl cyanide.^60^ The subsequent enzymatic kinetic resolution of the racemic Vince lactam with lactamase furnished the corresponding amino acid 113 (with (−) lactamase) or the (−) Vince lactam 112 (using (+) lactamase), which was then hydrolyzed into the desired amino acid 113.^61^ This key intermediate can then be converted into the desired product abacavir.^62^

The Vince lactam is not only a useful intermediate in the synthesis of abacavir, but also in the synthesis of peramivir and carbovir. In fact, numerous groups have reported the application of different lactamases for the resolution of the Vince lactam, en route to these powerful antiviral agents.^63^ A work carried out by Tardibono *et al.*^64^ presents a bio-click methodology towards homoabacavir 116b (Scheme 16b). In this approach, the hetero Diels Alder-reaction between cyclopentadiene and the transient nitroso-compound of *N*-Boc-hydroxylamine is used for the formation of intermediate 114. The subsequent reduction of the N–O bond furnished the racemic aminocyclopentenol derivative 115, which was then successfully subjected to a kinetic resolution with CALB lipase, using vinyl acetate with posterior basic hydrolysis, furnishing 115-(−) (80% ee) as a key intermediate for the further syntheses of homoabacavir 116b and homocarbovir.^64^

### 3.3 Epoxide-opening and biocatalysis

Epoxides are valuable and versatile building blocks and synthetic intermediates common in nature and synthesis.^65,66^ The outstanding reactivity of the three-membered ring is mainly caused by the special geometry and angle strain, endowing the ring opening reaction with a favourable thermodynamic driving force (24 to 28 kcal mol^−1^).^67,68^ The S_N_2 ring opening of epoxides shows a variety of desirable characteristics, such as reliability, stereospecificity and regioselectivity.^4^ The congener aziridines present similar characteristics, however with the advantage of the presence of a nitrogen atom, allowing subsequent chemical transformations in a somewhat easier way.^69,70^ Nevertheless, fewer efficient methods for the direct synthesis of aziridines are available,^71^ also reflected in the scarce number of respective bio-click approaches incorporating these substrates. The recent development of methods for the enzyme-catalyzed olefin aziridination^72^ could change these shortcomings and foster the development of a greater number of bio-click approaches incorporating aziridines.

The ring-opening of epoxides has been extensively applied for the synthesis of complex molecules.^73^ In fact, the synthesis of numerous Active Pharmaceutical Ingredients (APIs) uses ring-opening of epoxides as a key step.^74,75^ The bio-click approach towards the ring-opening reaction of epoxides is a field of increasing interest.

**Scheme 17:**
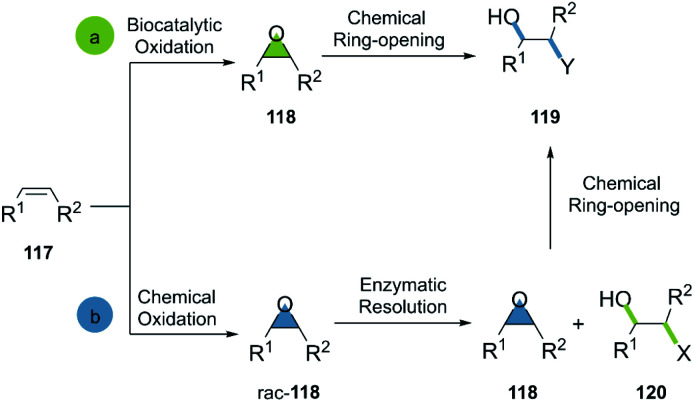
General bio-click approach in epoxide-opening reactions.

Generally, the bio-click approach incorporating epoxides is applied in two ways: (a) the selective enzymatic oxidation of alkenes, furnishing epoxides in green conditions for the subsequent ring opening; (b) the oxidation of alkenes, a subsequent enzymatic resolution of the racemic epoxides and the ring-opening of the desired candidate (Scheme 17).

#### 3.3.1 Epoxide-opening and oxidoreductases

The oxidoreductases are useful for the synthesis of enantiopure epoxides.^76^ Also, the enantioselective biooxidation of alkenes has been used as a key step in the bio-inspired synthesis of natural products.^77,78^ The directed evolution and rational design of enzymes has allowed the optimization of selectivity, catalytic efficiency and stability of oxidoreductases towards the levels necessary for industrial applications.^79,80^ Regarding the bio-click approach, the direct or indirect synthesis of epoxides using oxidoreductases is predominant. However, the biooxidation, chemical epoxidation and ring opening approach has been used in the total synthesis of molecules with multiple contiguous stereocenters.

**Scheme 18:**
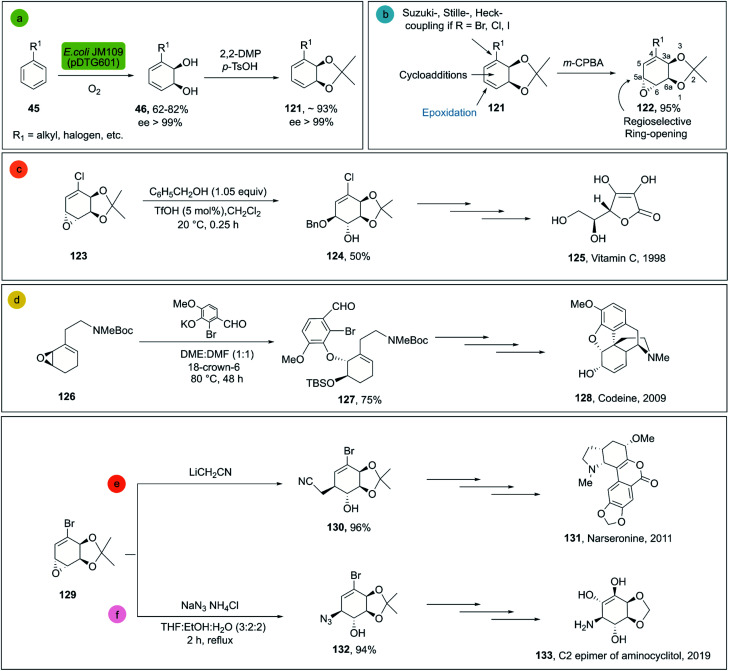
(a) Biocatalytic dihydroxylation of arenes by *E. coli* JM109 (pDT601), (b) chemical reactivity of *cis*-1,2-dihydrocatechols. Representative molecules obtained from bio-click methodologies (TDO-ring opening). (c) Vitamin C 1998 by Longmore *et al.*;^87^ (d) codeine 2009 by Leisch;^88^ (e) narseronine 2011 by Schwartz *et al.*;^89^ (f) C2 epimer of aminocyclitol 2019 by Carrau.^90^

*cis*-1,2-Dihydrocatechols 46 are obtained by the enzymatic dihydroxylation of arenes 45 and represent an excellent platform for the synthesis of alkaloids, sugars, cyclitols, prostaglandins, terpenes, polymers and others (Scheme 18a).^81^ As an example, the enantioselective synthesis of *cis*-1,2-dihydrocatechols is carried out in a whole-cell fermentation, which provides an efficient cofactor regeneration system.^82^*E. coli* JM109 (pDT601) which contains the genes from *Pseudomonas putida* F1 for the overexpression of the toluene dioxygenase system (TDO),^83^ is the most common biocatalyst applied for the enantioselective synthesis of *cis*-1,2-dihydrocatechol derivates. Careful optimization of the reaction conditions in a biphasic system can achieve yields as high as 35 g L^−1^.^84^ This is quite remarkable, considering that no efficient chemical method is known for the production of this class of compounds at scale.^79,81^

The *cis*-1,2-dihydrocatechols are highly versatile and a great platform for enantioselective synthesis^85^ (Scheme 18b). Monoepoxidation, for example, is a transformation that can be easily applied to *cis*-1,2-dihydrocatechols 121 and combined with enantio- and regioselective 122 ring-openings, which can be efficiently applied in total synthesis.^86^

For the ring opening of the epoxides on dihydrocatechols 122 the general preference is towards the cleavage in the 5a position (see Scheme 18b), mainly associated to two factors: (a) the greater stabilization of the transient carbocation in the allylic position; (b) less steric hindrance at position 5a as compared to 6a. In this way, the cleavage of epoxides is generally achieved with high regio- and enantioselectivity, providing the corresponding products of the *trans*-1,2 addition efficiently.^86^

Representative examples for the opening of monoepoxides 122 (obtained from biocatalytic enzymatic *cis*-dihydroxylation of benzene derivates 45) with different nucleophiles are ubiquitous (see Scheme 18, vitamin C,^87^ codeine,^88^ narseronine,^89^ C2 epimer of aminocyclitol^90^) and crucial in the synthesis of natural products.

The indirect epoxidation of α-haloketones using ketoreductases (KREDs) has become a common strategy to obtain chiral epoxides, also reflected in the stereoselective synthesis of different APIs obtained on a kg-scale. The development of new variants of KREDs through protein engineering has widely enhanced scope, robustness, and selectivity of these biocatalysts. The fact that they are an excellent green alternative to conventional catalysts is further underscored by the easy workup, generally avoiding chromatographic product purifications.

**Scheme 19:**
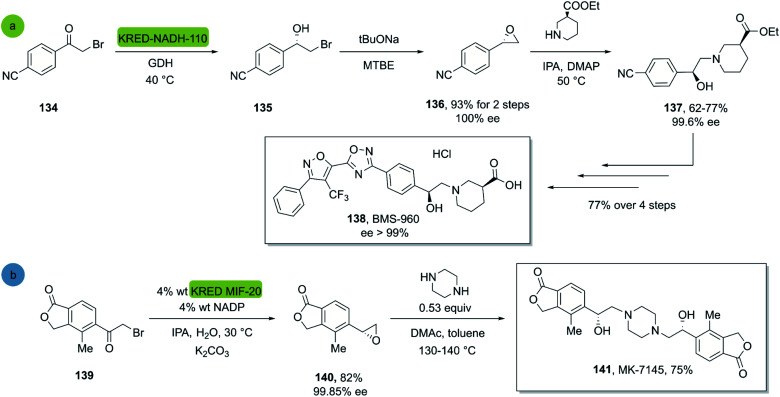
KRED mediated epoxidation of alfa-bromo ketones and ring opening: (a) synthesis of BMS-960, (b) synthesis of MK7145.

In 2017, Hou *et al.* reported a chemo-enzymatic synthesis of BMS960, a powerful S1P_1_-receptor agonist.^91^ Its synthesis involved the enzymatic reduction of α-bromoketone 134 (100 g in 5 h at 40 °C) to the corresponding alcohol 135 using the commercially available KRED-NADH-110 (substrate enzyme ratio 200 : 1). The chiral alcohol was then extracted with MTBE and reacted with sodium *tert*-butoxide towards the epoxide with 93% overall yield and 100% ee (*S*-enantiomer). Subsequently, a regio- and stereospecific ring opening of the epoxide 136 with (*S*)-ethyl piperidine-3-carboxilate in the presence of a catalytic amount of DMAP at 50 °C, provided the product 137 in up to 77% yield and with an ee of 99.6% after recrystallization. Four successive chemical transformations furnished BMS-960 in 23–33% overall yield (Scheme 19a).

In 2020, Ruck *et al.* reported a kg-scale synthesis of MK-7145, a clinical candidate for the treatment of hypertension and associated heart failures.^92^ The synthesis involved the enzymatic reduction of a bromo-ketone 139 to the corresponding bromohydrin as a key step. The commercial KRED-MIF-20-induced reduction and a subsequent ring closure furnished the corresponding enantiopure epoxide in 82% yield and excellent enantiopurity. Subsequently the target molecule MK-7145 141 was obtained through a bis-epoxide opening with piperazine at 140 °C, furnishing the product in 75% yield (Scheme 19b).

**Scheme 20:**
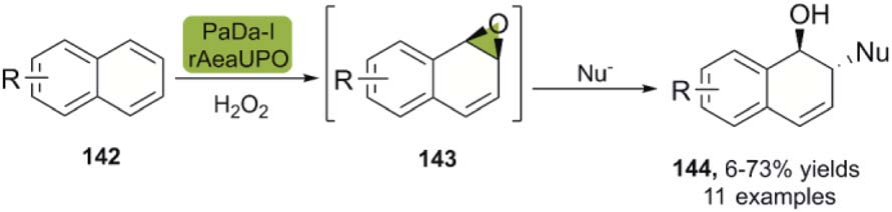
Bio-click synthesis of *trans*-disubstituted cyclohexadiene derivatives using peroxygenase PaDa-I, and chemical ring-opening with different nucleophiles.

Similar to the abovementioned biocatalytic *cis*-dihydroxylation of arenes 142, the Hollmann group recently described the direct biocatalytic epoxidation of naphthalene derivatives.^93^ The methodology described is based on the recombinantly evolved peroxygenase variant PaDa-I from *Agrocybe aegerita* (rAaeUPO), a self-sufficient enzyme. Careful control of the reaction time of the nucleophilic addition allows for the generation of the ring-opening products 144 using nucleophiles such as sodium azide (Scheme 20).

In this way, the synthesis of eleven derivatives in yields ranging from 6 to 73% was reported. These arene oxides are excellent building blocks for the synthesis of *trans*-disubstituted cyclohexadienes and can be further functionalized towards triazoles, amino alcohols, and other valuable entities. Epichlorohydrin 145 is a versatile molecule with multiple applications,^94^ and a useful starting material for the synthesis of different APIs.

**Scheme 21:**
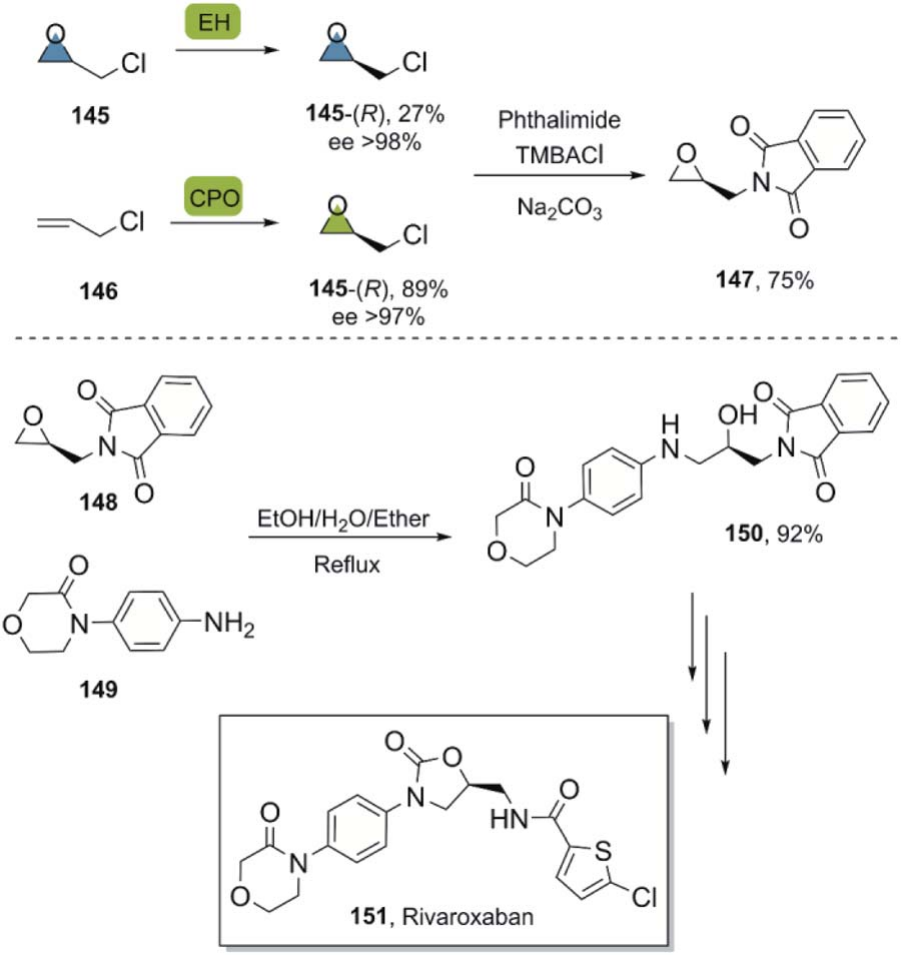
Bio-click approach for the synthesis of rivaroxaban.

The synthesis of rivaroxaban, an anticoagulant widely used in the prophylaxis of cardiovascular diseases,^95^ has been described by numerous groups using (*R*)-epichlorohydrin 145 as a building block.^96^ In a bio-click approach, rivaroxaban was synthesized *via* (*R*)-145, which can be obtained either by an enantioselective biooxidation of 3-chloropropene 146 using chloroperoxidase CPO from *Caldariomyces fumago*,^97^ or by resolution of racemic 145 using epoxyhydrolase EH ArEH^98^ from *Agrobacterium radiobacter* (Scheme 21). Further functionalisation of the desired epoxide with phthalimide under basic conditions using a phase transfer agent and a subsequent regio- and enantiospecific ring opening of epoxide 148 with arylamine 149, furnished the product 150 smoothly en route to rivaroxaban.^99^

#### 3.3.2 Epoxide-opening and hydrolases

**Scheme 22:**
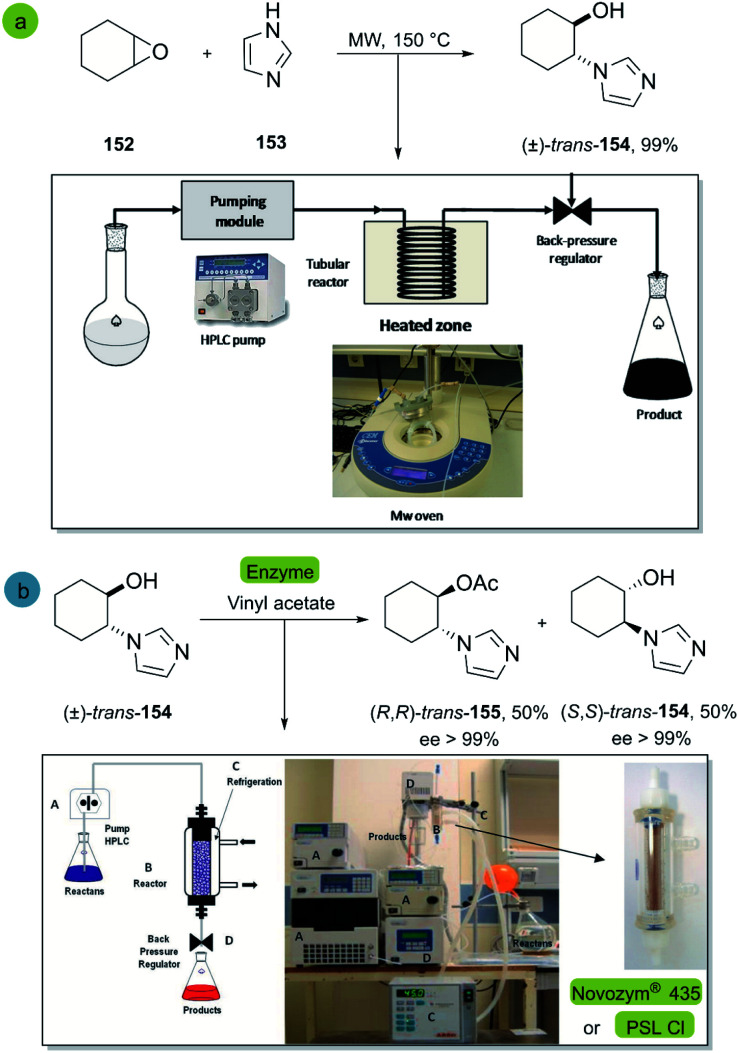
Synthesis of *trans*-2-(1*H*-imidazol-1-yl)cyclohexanol (b) enantioselective acylation of (±)-*trans*-2-(1*H*-imidazol-1-yl)cyclohexanol 154 with vinyl acetate catalyzed by Novozym® 435 or PSL-CI. This figure was used with permission.^100^

In 2012, Porcar *et al.*^100^ reported the stereoselective chemoenzymatic synthesis of imidazoles under continuous flow conditions for the generation of new chiral ionic liquids.^101^ The first step is the epoxide opening of cyclohexene oxide 152 with imidazole 153 in a continuous flow reactor with microwave (MW) irradiation (Scheme 22). The resulting cyclohexanol derivative (±)-*trans*-154 is then subjected to the second step, an acylation with vinyl acetate catalyzed by the commercially available immobilized lipases Novozym® 435 or PSL-CI (*Pseudomonas cepacia* lipase), allowing an efficient kinetic resolution. The applied continuous-flow methodology demonstrated high efficiency in a batch process, and the lipases provided the corresponding acylated kinetically-resolved product 155 with excellent enantioselectivities (>99%) and virtually complete conversion.

**Scheme 23:**
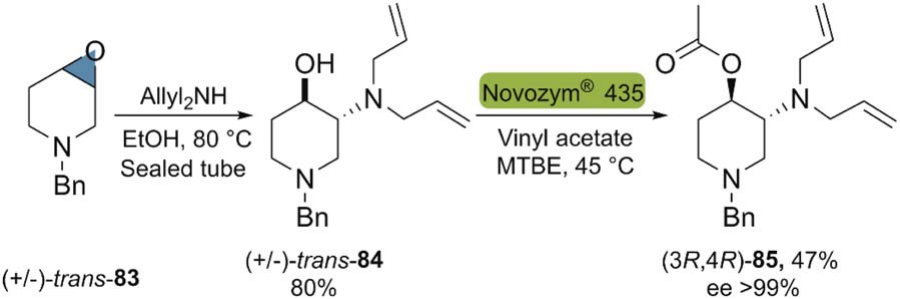
Chemoenzymatic synthesis of optically pure orthogonally protected t*rans*-3-amino-4-hydroxypiperidines.

In 2015, Villar *et al.* reported the chemoenzymatic synthesis of optically pure, orthogonally protected *trans*-3-amino-4-hydroxypiperidines, potential molecules for the synthesis of chiral bioactive compounds (Scheme 23).^102^ The developed bio-click methodology developed, starts with the regioselective epoxide-opening with diallylamine, providing access to the racemic *trans*-cyclohexanol 157. The subsequent enzymatic kinetic resolution *via* transesterification with vinyl acetate was performed with Novozym® 435, which gave the desired product 158 with the highest conversion (47%) and enantioselectivity (ee > 99%) among the enzymes tested.

**Scheme 24:**
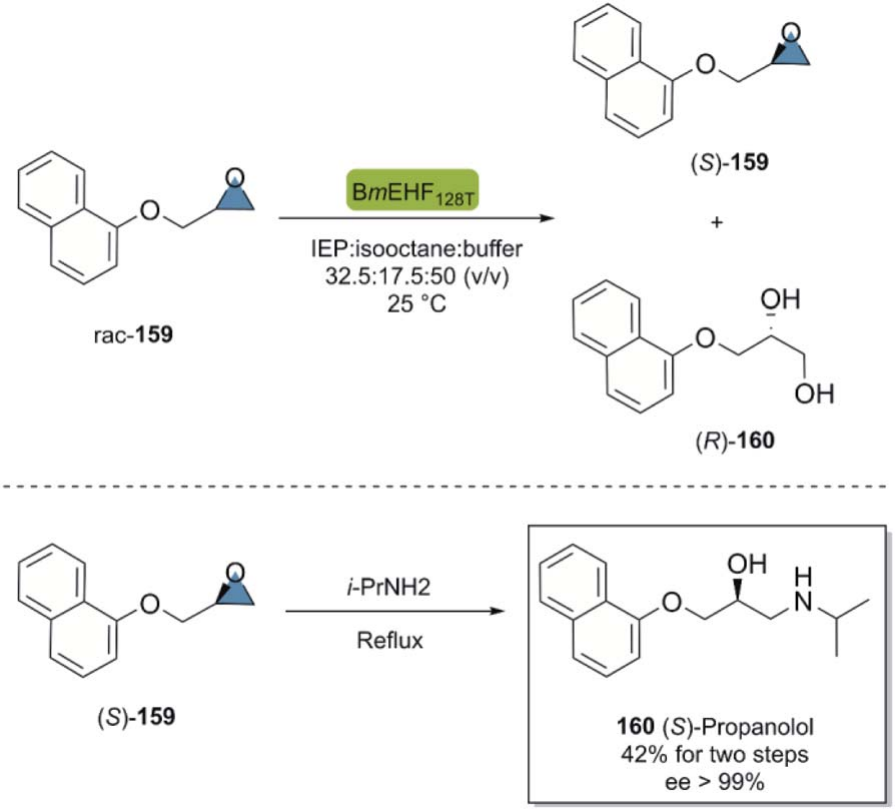
Chemoenzymatic synthesis of (*S*)-propranolol.

Propranolol is a versatile beta-adrenergic receptor antagonist used in the treatment of several cardiovascular such as hypertension, cardiac arrhythmias, *etc.*^103^ Quite remarkably, (*S*)-propranolol is 100 times more potent β-adrenergic receptor blocker than its enantiomer.^104^ In 2015, Dong *et al.*^105^ reported the chemoenzymatic two-step synthesis of (*S*)-propranolol (Scheme 24). The enzymatic kinetic resolution of racemic epoxide 159 with the hydrolase BmEH128T (from *Bacillus megaterium*), smoothly furnished the (*S*)-configured product 159 smoothly after centrifugation of the undesired enantiomer. The subsequent regioselective ring-opening of (*S*)-159 with isopropylamine under reflux conditions gave (*S*)-propanolol 160 in an overall yield of 42% and excellent enantioselectivity after recrystallization (ee > 99%).

### 3.4 Thiol-Michael reactions and biocatalysis

**Scheme 25:**
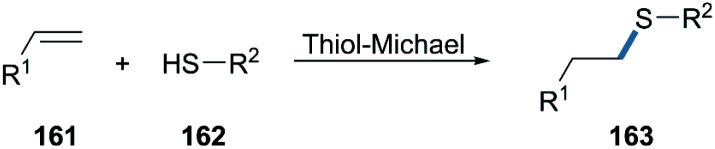
Thiol-Michael click chemistry.

Thiol-Michael click reactions, either *via* free-radical, or catalyzed Michael-additions, are valuable transformations widely recognized in polymer science and bioconjugation chemistry, since they allow for the efficient formation of C–S bonds from thiols and alkenes (Scheme 25).^106–108^ They are usually highly efficient, work under mild reaction conditions, are regioselective and atom-economic, and only generate few or no by-products which are generally easily separated.^109,110^ Bio-click reactions approaches featuring thiol-Michael chemistry are scarce to date, despite the advantages of chemoenzymatic reactions in this context.

#### 3.4.1 Thiol-Michael reactions and oxidoreductases

**Scheme 26:**
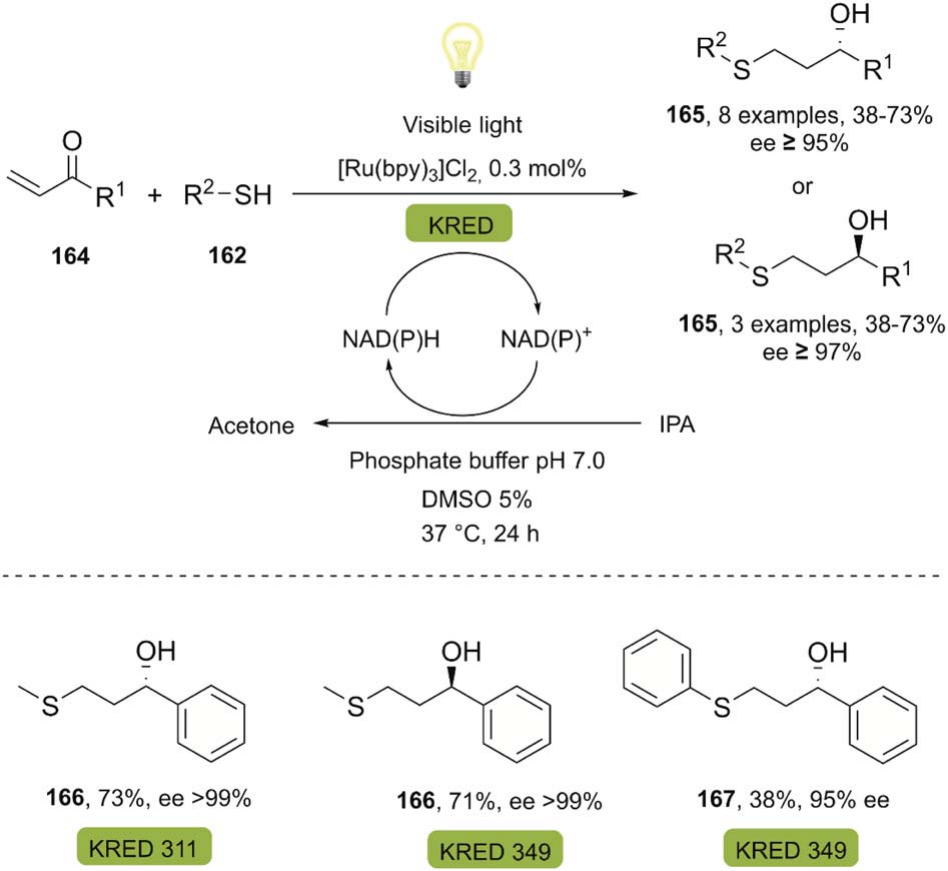
One-pot photo-biocatalytic (thiol-Michael/KRED reduction).

The spatial and temporal control of light as an energy source for thiol-Michael click reactions proved valuable in tandem bio-click reactions, as reported in 2018 by Lauder *et al.* for the synthesis of volatile chiral 1,3-mercaptoalkanols 166–167.^111^ The reported one-pot methodology comprises a thiol-Michael reaction photocatalyzed by visible light combined with [Ru(bpy)_3_]Cl_2_ (0.3 mol%), fully compatible with the conditions of the subsequent bioreduction using KREDs (see Scheme 26). The enzymes used furnished the corresponding (*S*)- or (*R*)-enantiomers in yields (38–73%) and high enantiomeric excess.

## 4. Summary and outlook

This review summarizes developments in a field of research that combines biocatalytic transformations and click reactions. The proposal of the term bio-click chemistry comes quite naturally if one considers the varied and efficient syntheses of complex molecules explored. A clear classification of the different chemoenzymatic transformations will undoubtedly promote the development of more sustainable and efficient processes in this area (see Table 1).

**Table 1:** Representative molecules obtained *via* bio-click chemistry

Click reaction	Enzyme or microorganism	Structure	Function	Reference
CuAAC	Carbonyl reductase from *Candida magnoliae* (CMCR)	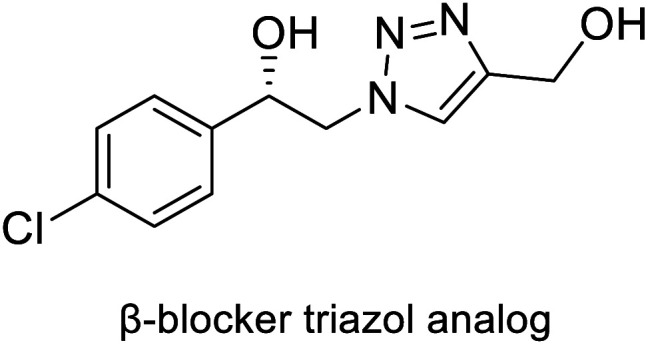	β-Adrenergic receptor blocker analogue	29
Diels–Alder or hetero-Diels–Alder	(+)-γ-Lactamase, (−)-γ-lactamase from *Bradyrhizobium japonicum* USDA 6	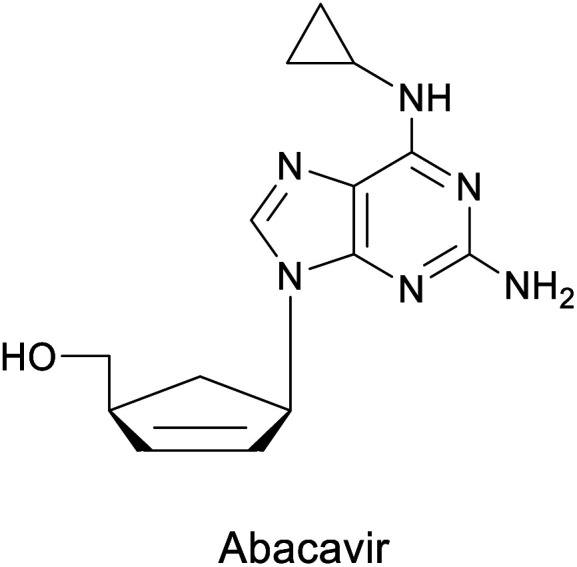	Carbocyclic nucleoside with antiviral activity	58, 60, 61 and 112
	*Candida antarctica* B lipase (CALB)			64
	Oxidoreductase SorbC	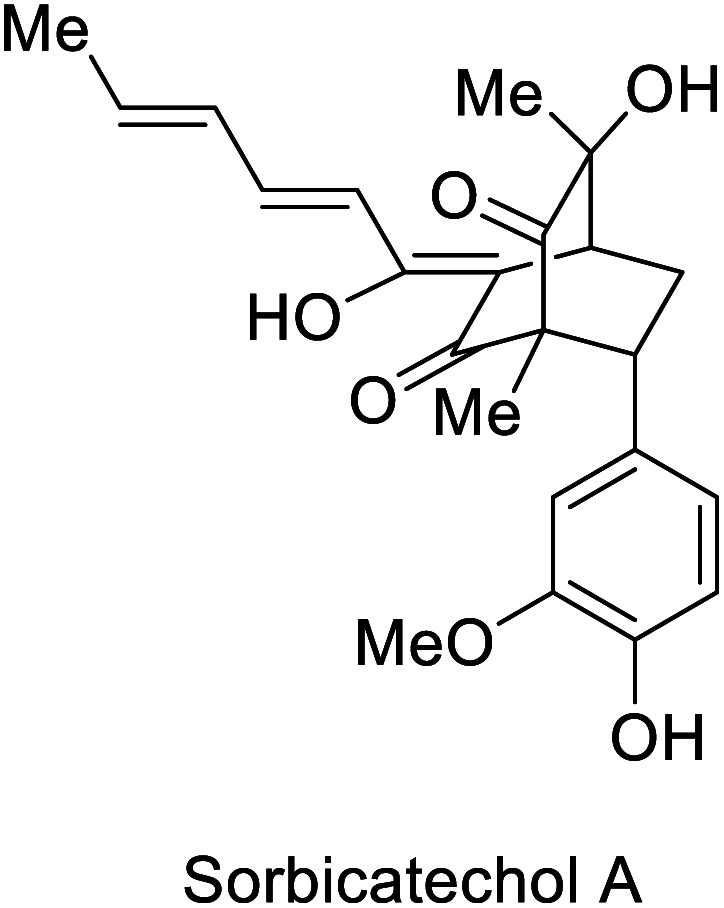	Antiviral activity against H1N1	53, 54 and 113
Epoxide-opening	*E. coli* JM109 (pDTG601)	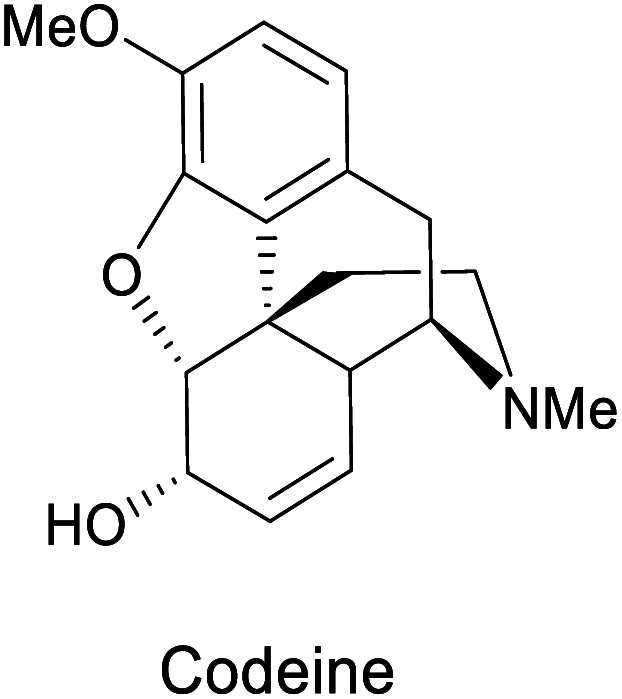	Painkiller	88
	Ketoreductase, KRED-NADH-110	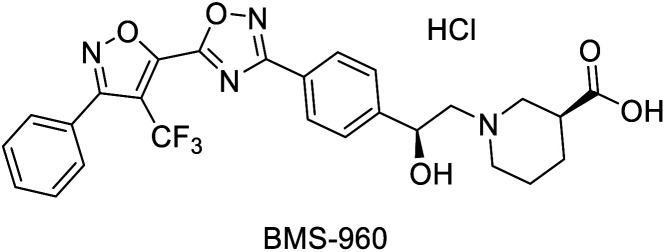	S1P1 receptor agonist	91
Thiol-Michael	Ketoreductase, KRED-311	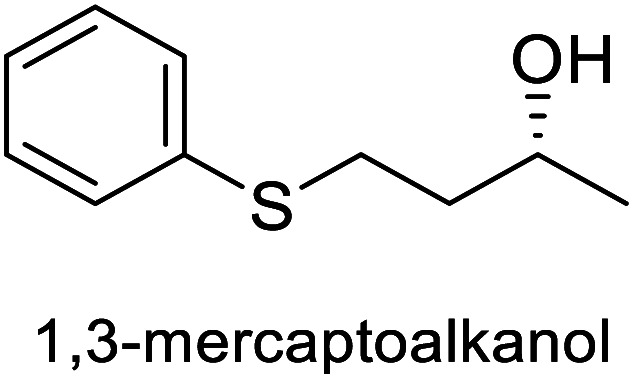	Volatile sulfur compound	111

Bio-click chemistry reactions are demonstrated to be remarkably complementary, as is the case of click chemical reactions, which are regiospecific by nature but not necessarily enantioselective. The joint use of enzymes/microorganisms allows to overcome these limitations, introducing chirality without hampering the overall efficiency of the processes. The global need of, and tendency towards more sustainable and green processes is thoroughly reflected by the complementary use of efficient chemical reactions and green biocatalysis. It can be envisioned, that the development of more robust and active biocatalysts with wider substrate ranges will facilitate the broadening of efficient bio-click processes foreseeable future. A very promising example are ketoreductases (KREDs), which are by now widely commercially available in great variety. The use of KREDs has proven to be a competitive alternative to conventional enantioselective oxidations, even on an industrial scale.

It is essential to emphasize, that click reactions, biocatalysis or their combination do not generate greener processes *per se*, and it is always necessary to carefully evaluate the exact methodology, the implementation in a reaction sequence and the expected overall efficiency and sustainability for future improvements.

## Conflicts of interest

There are no conflicts to declare.

## Supplementary Material

## References

[cit1] Hönig M., Sondermann P., Turner N. J., Carreira E. M. (2017). Angew. Chem., Int. Ed..

[cit2] Bornscheuer U. T., Huisman G. W., Kazlauskas R. J., Lutz S., Moore J. C., Robins K. (2012). Nature.

[cit3] Cortes-Clerget M., Akporji N., Zhou J., Gao F., Guo P., Parmentier M., Gallou F., Berthon J.-Y., Lipshutz B. H. (2019). Nat. Commun..

[cit4] Kolb H. C., Finn M. G., Sharpless K. B. (2001). Angew. Chem., Int. Ed..

[cit5] Erythropel H. C., Zimmerman J. B., de Winter T. M., Petitjean L., Melnikov F., Lam C. H., Lounsbury A. W., Mellor K. E., Janković N. Z., Tu Q., Pincus L. N., Falinski M. M., Shi W., Coish P., Plata D. L., Anastas P. T. (2018). Green Chem..

[cit6] Oueis E., Sabot C., Renard P.-Y. (2015). Chem. Commun..

[cit7] Palomo J. M. (2013). Curr. Org. Chem..

[cit8] Brennan J. L., Hatzakis N. S., Tshikhudo T. R., Razumas V., Patkar S., Vind J., Svendsen A., Nolte R. J. M., Rowan A. E., Brust M. (2006). Bioconjugate Chem..

[cit9] Palomo J. M. (2012). Org. Biomol. Chem..

[cit10] Woodward R. B., Ayer W. A., Beaton J. M., Bickelhaupt F., Bonnett R., Buchschacher P., Closs G. L., Dutler H., Hannah J., Hauck F. P., Itô S., Langemann A., Le Goff E., Leimgruber W., Lwowski W., Sauer J., Valenta Z., Volz H. (1960). J. Am. Chem. Soc..

[cit11] Nicolaou K. C., Yang Z., Liu J. J., Ueno H., Nantermet P. G., Guy R. K., Claiborne C. F., Renaud J., Couladouros E. A., Paulvannan K., Sorensen E. J. (1994). Nature.

[cit12] Holton R. A., Kim H. B., Somoza C., Liang F., Biediger R. J., Boatman P. D., Shindo M., Smith C. C., Kim S. (1994). J. Am. Chem. Soc..

[cit13] Durek T., Torbeev V. Y., Kent S. B. H. (2007). Proc. Natl. Acad. Sci. U. S. A..

[cit14] Noyori R. (2009). Nat. Chem..

[cit15] Thirumurugan P., Matosiuk D., Jozwiak K. (2013). Chem. Rev..

[cit16] Prasher P., Sharma M. (2019). Medchemcomm.

[cit17] Iha R. K., Wooley K. L., Nyström A. M., Burke D. J., Kade M. J., Hawker C. J. (2009). Chem. Rev..

[cit18] Tasdelen M. A., Kiskan B., Yagci Y. (2016). Prog. Polym.
Sci..

[cit19] Sheldon R. A., Woodley J. M. (2018). Chem. Rev..

[cit20] Martinez C. A., Hu S., Dumond Y., Tao J., Kelleher P., Tully L. (2008). Org. Process Res. Dev..

[cit21] Sheldon R. A. (2016). Green Biocatal..

[cit22] Huffman M. A., Fryszkowska A., Alvizo O., Borra-Garske M., Campos K. R., Canada K. A., Devine P. N., Duan D., Forstater J. H., Grosser S. T., Halsey H. M., Hughes G. J., Jo J., Joyce L. A., Kolev J. N., Liang J., Maloney K. M., Mann B. F., Marshall N. M., McLaughlin M., Moore J. C., Murphy G. S., Nawrat C. C., Nazor J., Novick S., Patel N. R., Rodriguez-Granillo A., Robaire S. A., Sherer E. C., Truppo M. D., Whittaker A. M., Verma D., Xiao L., Xu Y., Yang H. (2019). Science.

[cit23] Leung M. K. M., Hagemeyer C. E., Johnston A. P. R., Gonzales C., Kamphuis M. M. J., Ardipradja K., Such G. K., Peter K., Caruso F. (2012). Angew. Chem., Int. Ed..

[cit24] Blackman M. L., Royzen M., Fox J. M. (2008). J. Am. Chem. Soc..

[cit25] Dong J., Krasnova L., Finn M. G., Sharpless K. B. (2014). Angew. Chem., Int. Ed..

[cit26] Meng G., Guo T., Ma T., Zhang J., Shen Y., Sharpless K. B., Dong J. (2019). Nature.

[cit27] Meghani N. M., Amin H. H., Lee B.-J. (2017). Drug Discovery Today.

[cit28] Brik A., Alexandratos J., Lin Y.-C., Elder J. H., Olson A. J., Wlodawer A., Goodsell D. S., Wong C.-H. (2005). ChemBioChem.

[cit29] Ankati H., Yang Y., Zhu D., Biehl E. R., Hua L. (2008). J. Org. Chem..

[cit30] Aguirre-Pranzoni C., Tosso R. D., Bisogno F. R., Kurina-Sanz M., Orden A. A. (2019). Process Biochem..

[cit31] Sivaguru P., Ning Y., Bi X. (2021). Chem. Rev..

[cit32] Szymanski W., Postema C. P., Tarabiono C., Berthiol F., Campbell-Verduyn L., de Wildeman S., de Vries J. G., Feringa L., Janssen D. B. (2010). Adv. Synth. Catal..

[cit33] Campbell-Verduyn L. S., Mirfeizi L., Dierckx R. A., Elsinga P. H., Feringa L. (2009). Chem. Commun..

[cit34] Nguyen T. N., Chen P.-A., Setthakarn K., May J. A. (2018). Molecules.

[cit35] Leijondahl K., Borén L., Braun R., Bäckvall J.-E. (2008). Org. Lett..

[cit36] Cuetos A., Bisogno F. R., Lavandera I., Gotor V. (2013). Chem. Commun..

[cit37] de Souza de Oliveira C., de Andrade K. T., Omori A. T. (2013). J. Mol. Catal. B: Enzym..

[cit38] de la Sovera V., Bellomo A., Pena J. M., Gonzalez D., Stefani H. A. (2011). Mol. Diversity.

[cit39] Sabatini M. T., Boulton L. T., Sneddon H. F., Sheppard T. D. (2019). Nat. Catal..

[cit40] Pedersen D. S., Abell A. (2011). Eur. J. Org. Chem..

[cit41] Hassan S., Tschersich R., Müller T. J. J. (2013). Tetrahedron Lett..

[cit42] Gesse P., Müller T. J. J. (2019). Eur. J. Org. Chem..

[cit43] Büyükadali N. N., Seven S., Aslan N., Yenidede D., Gümüş A. (2015). Tetrahedron: Asymmetry.

[cit44] Moisă M. E., Poppe L., Gal C. A., Bencze L. C., Irimie F. D., Paizs C., Peter F., Toşa M. I. (2018). React. Chem. Eng..

[cit45] Tasdelen M. A. (2011). Polym. Chem..

[cit46] Otto S., Blokzijl W., Engberts J. B. F. N. (1994). J. Org. Chem..

[cit47] Wassermann A. (1942). J. Chem. Soc..

[cit48] Otto S., Engberts J. B. F. N. (1995). Tetrahedron Lett..

[cit49] Vermeeren P., Hamlin T. A., Fernández I., Bickelhaupt F. M. (2020). Angew. Chem., Int. Ed..

[cit50] Meng J., Wang X., Xu D., Fu X., Zhang X., Lai D., Zhou L., Zhang G. (2016). Molecules.

[cit51] Sib A., Gulder T. A. M. (2017). Angew. Chem., Int. Ed..

[cit52] Hong R., Chen Y., Deng L. (2005). Angew. Chem., Int. Ed..

[cit53] Sib A., Gulder T. A. M. (2018). Angew. Chem., Int. Ed..

[cit54] Baker Dockrey S. A., Lukowski A. L., Becker M. R., Narayan A. R. H. (2018). Nat. Chem..

[cit55] Caille J.-C., Govindan C. K., Junga H., Lalonde J., Yao Y. (2002). Org. Process Res. Dev..

[cit56] Jirousek M. R., Gillig J. R., Gonzalez C. M., Heath W. F., McDonald J. H., Neel D. A., Rito C. J., Singh U., Stramm L. E., Melikian-Badalian A., Baevsky M., Ballas L. M., Hall S. E., Winneroski L. L., Faul M. M. (1996). J. Med. Chem..

[cit57] Wirz B., Spurr P., Pfleger C. (2010). Tetrahedron: Asymmetry.

[cit58] Moyle G. J., DeJesus E., Cahn P., Castillo S. A., Zhao H., Gordon D. N., Craig C., Scott T. R. (2005). JAIDS, J. Acquired Immune Defic. Syndr..

[cit59] Crimmins M. T., King B. W. (1996). J. Org. Chem..

[cit60] Singh R., Vince R. (2012). Chem. Rev..

[cit61] Zhu S., Ren L., Yu S., Gong C., Song D., Zheng G. (2014). Bioorg. Med. Chem. Lett..

[cit62] Daluge S. M., Martin M. T., Sickles B. R., Livingston D. A. (2000). Nucleosides, Nucleotides Nucleic Acids.

[cit63] Slagman S., Fessner W.-D. (2021). Chem. Soc. Rev..

[cit64] Tardibono L. P., Miller M. J., Balzarini J. (2011). Tetrahedron.

[cit65] Zou Y., Garcia-Borràs M., Tang M. C., Hirayama Y., Li D. H., Li L., Watanabe K., Houk K. N., Tang Y. (2017). Nat. Chem. Biol..

[cit66] Bonini C., Righi G. (2002). Tetrahedron.

[cit67] Pellissier H. , Lattanzi A. and Dalpozzo R., in Asymmetric Synthesis of Three-Membered Rings, Wiley-VCH Verlag GmbH & Co. KGaA, Germany, 1st edn, 2017, ch. 3, pp. 379–538

[cit68] Morgan K. M., Ellis J. A., Lee J., Fulton A., Wilson S. L., Dupart P. S., Dastoori R. (2013). J. Org. Chem..

[cit69] Hu X. E. (2004). Tetrahedron.

[cit70] Lu P. (2010). Tetrahedron.

[cit71] Pellissier H. , Lattanzi A. and Dalpozzo R., in Asymmetric Synthesis of Three-Membered Rings, Wiley-VCH Verlag GmbH & Co. KGaA, Germany, 1st edn, 2017, ch. 2, pp. 205–378

[cit72] Farwell C. C., Zhang R. K., McIntosh J. A., Hyster T. K., Arnold F. H. (2015). ACS Cent. Sci..

[cit73] Crotti P. and Pineschi M., in Aziridines Epoxides Organic Synthesis, ed. A.K. Yudin, Wiley-VCH, Weinheim, 1st edn.2006, pp. 271–313

[cit74] Russell M. G., Jamison T. F. (2019). Angew. Chem., Int. Ed..

[cit75] Moschona F., Savvopoulou I., Tsitopoulou M., Tataraki D., Rassias G. (2020). Catal..

[cit76] Archelas A., Furstoss R. (1997). Annu. Rev. Microbiol..

[cit77] Archelas A., Furstoss R. (1992). Tetrahedron Lett..

[cit78] Lakner F. J., Hager L. P. (1996). J. Org. Chem..

[cit79] Martínez A. T., Ruiz-Dueñas F. J., Camarero S., Serrano A., Linde D., Lund H., Vind J., Tovborg M., Herold-Majumdar O. M., Hofrichter M., Liers C., Ullrich R., Scheibner K., Sannia G., Piscitelli A., Pezzella C., Sener M. E., Kılıç S., van Berkel W. J. H., Guallar V., Lucas M. F., Zuhse R., Ludwig R., Hollmann F., Fernández-Fueyo E., Record E., Faulds C. B., Tortajada M., Winckelmann I., Rasmussen J.-A., Gelo-Pujic M., Gutiérrez A., del Río J. C., Rencoret J., Alcalde M. (2017). Biotechnol. Adv..

[cit80] Sheldon R. A., Brady D. (2019). ChemSusChem.

[cit81] Hudlicky T. (2018). ACS Omega.

[cit82] Özgen F. F. and Schmidt S., in Biocatalysis. Enzymatic Basics and Applications, ed. Q. Husain and M. F. Ullah, Springer International Publishing, New York, 1st edn, 2019, ch. 4, pp. 57–82

[cit83] Zylstra G. J., McCombie W. R., Gibson D. T., Finette B. A. (1988). Appl. Environ. Microbiol..

[cit84] Vila M. A., Brovetto M., Gamenara D., Bracco P., Zinola G., Seoane G., Rodríguez S., Carrera I. (2013). J. Mol. Catal. B: Enzym..

[cit85] Hudlicky T., Price J. D., Rulin F., Tsunoda T. (1990). J. Am. Chem. Soc..

[cit86] Banwell M. G., Haddad N., Hudlicky T., Nugent T. C., Mackay M. F., Richards S. L. (1997). J. Chem. Soc., Perkin Trans. 1.

[cit87] Banwell M., Blakey S., Harfoot G., Longmore R. (1998). J. Chem. Soc., Perkin Trans. 1.

[cit88] Leisch H., Omori A. T., Finn K. J., Gilmet J., Bissett T., Ilceski D., Hudlický T. (2009). Tetrahedron.

[cit89] Schwartz B. D., Banwell M. G., Cade I. A. (2011). Tetrahedron Lett..

[cit90] Carrau G., Bellomo A. I., Suescun L., Gonzalez D. (2019). Eur. J. Org. Chem..

[cit91] Hou X., Zhang H., Chen B.-C., Guo Z., Singh A., Goswami A., Gilmore J. L., Sheppeck J. E., Dyckman A. J., Carter P. H., Mathur A. (2017). Org. Process Res. Dev..

[cit92] Ruck R. T., Chen Q., Rivera N., Kong J., Mangion I. K., Tan L., Fleitz F. J. (2021). Org. Process Res. Dev..

[cit93] Zhang W., Li H., Younes S. H. H., Gómez de Santos P., Tieves F., Grogan G., Pabst M., Alcalde M., Whitwood A. C., Hollmann F. (2021). ACS Catal..

[cit94] Kasai N., Suzuki T., Furukawa Y. (1998). J. Mol. Catal. B: Enzym..

[cit95] Perzborn E., Roehrig S., Straub A., Kubitza D., Misselwitz F. (2010). Nat. Rev. Drug Discovery.

[cit96] Fattah T. A., Saeed A. (2017). Tetrahedron: Asymmetry.

[cit97] Wu J., Liu C., Jiang Y., Hu M., Li S., Zhai Q. (2010). Catal. Commun..

[cit98] Jin H.-X., Liu Z.-Q., Hu Z.-C., Zheng Y.-G. (2013). Eng. Life Sci..

[cit99] Mali A. C., Deshmukh D. G., Joshi D. R., Lad H. D., Patel P. I., Medhane V. J., Mathad V. T. (2015). Sustainable Chem. Processes.

[cit100] Porcar R., Sans V., Ríos-Lombardía N., Gotor-Fernández V., Gotor V., Burguete M. I., García-Verdugo E., V Luis S. (2012). ACS Catal..

[cit101] Busto E., Gotor-Fernández V., Ríos-Lombardía N., García-Verdugo E., Alfonso I., García-Granda S., Menéndez-Velázquez A., Burguete M. I., V Luis S., Gotor V. (2007). Tetrahedron Lett..

[cit102] Villar-Barro Á., Gotor V., Brieva R. (2015). Tetrahedron.

[cit103] V Srinivasan A. (2019). Ann. Indian Acad. Neurol..

[cit104] Silber B., Holford N. H. G., Riegelman S. (1982). J. Pharm. Sci..

[cit105] Kong X.-D., Yu H.-L., Yang S., Zhou J., Zeng B.-B., Xu J.-H. (2015). J. Mol. Catal. B: Enzym..

[cit106] Park E. J., Gevrek T. N., Sanyal R., Sanyal A. (2014). Bioconjugate Chem..

[cit107] Ghosh S. S., Kao P. M., McCue A. W., Chappelle H. L. (1990). Bioconjugate Chem..

[cit108] Nair D. P., Podgórski M., Chatani S., Gong T., Xi W., Fenoli C. R., Bowman C. N. (2014). Chem. Mater..

[cit109] Hoyle C. E., Bowman C. N. (2010). Angew. Chem., Int. Ed..

[cit110] Skinner E. K., Whiffin F. M., Price G. J. (2012). Chem. Commun..

[cit111] Lauder K., Toscani A., Qi Y., Lim J., Charnock S. J., Korah K., Castagnolo D. (2018). Angew. Chem., Int. Ed..

[cit112] Vince R., Hua M. (2006). Curr. Protoc. Nucleic Acid Chem..

[cit113] Peng J., Zhang X., Du L., Wang W., Zhu T., Gu Q., Li D. (2014). J. Nat. Prod..

